# The Population History of Domestic Sheep Revealed by Paleogenomes

**DOI:** 10.1093/molbev/msae158

**Published:** 2024-10-22

**Authors:** Damla Kaptan, Gözde Atağ, Kıvılcım Başak Vural, Pedro Morell Miranda, Ali Akbaba, Eren Yüncü, Aleksey Buluktaev, Mohammad Foad Abazari, Sevgi Yorulmaz, Duygu Deniz Kazancı, Ayça Küçükakdağ Doğu, Yasin Gökhan Çakan, Rana Özbal, Fokke Gerritsen, Bea De Cupere, Refik Duru, Gülsün Umurtak, Benjamin S Arbuckle, Douglas Baird, Özlem Çevik, Erhan Bıçakçı, Can Yumni Gündem, Evangelia Pişkin, Lamys Hachem, Kayra Canpolat, Zohre Fakhari, Maria Ochir-Goryaeva, Viktoria Kukanova, Hamid Reza Valipour, Javad Hoseinzadeh, Fatma Küçük Baloğlu, Anders Götherström, Eleftherios Hadjisterkotis, Thierry Grange, Eva-Maria Geigl, İnci Z Togan, Torsten Günther, Mehmet Somel, Füsun Özer

**Affiliations:** Department of Biological Sciences, Middle East Technical University, Ankara 06800, Turkey; Department of Biological Sciences, Middle East Technical University, Ankara 06800, Turkey; Department of Biological Sciences, Middle East Technical University, Ankara 06800, Turkey; Human Evolution Program, Department of Organismal Biology, Uppsala University, Uppsala, Sweden; Selçuklu ve Malazgirt Araştırma ve Uygulama Merkezi, Muş Alparslan Üniversitesi, Muş, Turkey; Department of Biological Sciences, Middle East Technical University, Ankara 06800, Turkey; Department of Archaeology, Ethnology and History, Kalmyk Scientific Center of the Russian Academy of Sciences, Elista, Russia; Division of Medical Sciences, Island Medical Program, University of British Columbia, Vancouver, BC, Canada; Department of Biology, Centre for Biomedical Research, University of Victoria, Victoria, Canada; Department of Biological Sciences, Middle East Technical University, Ankara 06800, Turkey; Department of Biological Sciences, Middle East Technical University, Ankara 06800, Turkey; Department of Biological Sciences, Middle East Technical University, Ankara 06800, Turkey; Department of Prehistory, Istanbul University, Laleli, Istanbul, Turkey; Department of Archaeology and History of Art, Koç University, Istanbul, Turkey; Netherlands Institute in Turkey, Istanbul, Turkey; Leiden Institute for Area Studies, Leiden University, Leiden, Netherlands; Operational Directorate Earth and History of Life, Royal Belgian Institute of Natural Sciences, Brussels, Belgium; Faculty of Letters, Department of Archaeology, İstanbul University, Laleli, Istanbul, Turkey; Faculty of Letters, Department of Archaeology, İstanbul University, Laleli, Istanbul, Turkey; Department of Anthropology, University of North Carolina at Chapel Hill, Chapel Hill, NC, USA; Department of Archaeology, Classics, and Egyptology, University of Liverpool, Liverpool, UK; Department of Archaeology, Trakya University, Edirne, Turkey; Department of Prehistory, Istanbul University, Laleli, Istanbul, Turkey; Department of Archaeology, Batman University, Batman, Turkey; Department of Settlement Archaeology, Middle East Technical University, Ankara, Turkey; Institut National de Recherches Archéologiques Préventives (Inrap), UMR 8215 Trajectoires, Paris, France; Department of Biological Sciences, Middle East Technical University, Ankara 06800, Turkey; Department of Biological Sciences, Middle East Technical University, Ankara 06800, Turkey; Department of Archaeology, Ethnology and History, Kalmyk Scientific Center of the Russian Academy of Sciences, Elista, Russia; Khalikov Institute of Archaeology, Academy of Sciences of Tatarstan, Kazan, The Republic of Tatarstan, Russia; Department of Archaeology, Ethnology and History, Kalmyk Scientific Center of the Russian Academy of Sciences, Elista, Russia; Department of Archaeology, Faculty of Letters and Human Sciences, Shahid Beheshti University, Tehran, Iran; Department of Archaeology, University of Kashan, Kashan, Iran; Department of Biology, Giresun University, Giresun, Turkey; Human-G Laboratory, Department of Anthropology, Hacettepe University, Beytepe, Ankara 06800, Turkey; Center for Paleogenetics, Stockholm, Sweden; Archaeological Research Laboratory, Department of Archaeology and Classical Studies, University of Stockholm, Stockholm, Sweden; Natural Resources and Environment, Agricultural Research Institute, Nicosia, Cyprus; Université de Paris, Institut Jacques Monod, CNRS, Paris, France; Université de Paris, Institut Jacques Monod, CNRS, Paris, France; Department of Biological Sciences, Middle East Technical University, Ankara 06800, Turkey; Human Evolution Program, Department of Organismal Biology, Uppsala University, Uppsala, Sweden; Department of Biological Sciences, Middle East Technical University, Ankara 06800, Turkey; Department of Anthropology, Hacettepe University, Ankara 06800, Turkey

**Keywords:** domestication, paleogenetics, sheep, ancient DNA, Mouflon, whole-genome sequencing, introgression

## Abstract

Sheep was one of the first domesticated animals in Neolithic West Eurasia. The zooarchaeological record suggests that domestication first took place in Southwest Asia, although much remains unresolved about the precise location(s) and timing(s) of earliest domestication, or the post-domestication history of sheep. Here, we present 24 new partial sheep paleogenomes, including a 13,000-year-old Epipaleolithic Central Anatolian wild sheep, as well as 14 domestic sheep from Neolithic Anatolia, two from Neolithic Iran, two from Neolithic Iberia, three from Neolithic France, and one each from Late Neolithic/Bronze Age Baltic and South Russia, in addition to five present-day Central Anatolian Mouflons and two present-day Cyprian Mouflons. We find that Neolithic European, as well as domestic sheep breeds, are genetically closer to the Anatolian Epipaleolithic sheep and the present-day Anatolian and Cyprian Mouflon than to the Iranian Mouflon. This supports a Central Anatolian source for domestication, presenting strong evidence for a domestication event in SW Asia outside the Fertile Crescent, although we cannot rule out multiple domestication events also within the Neolithic Fertile Crescent. We further find evidence for multiple admixture and replacement events, including one that parallels the Pontic Steppe-related ancestry expansion in Europe, as well as a post-Bronze Age event that appears to have further spread Asia-related alleles across global sheep breeds. Our findings mark the dynamism of past domestic sheep populations in their potential for dispersal and admixture, sometimes being paralleled by their shepherds and in other cases not.

## Introduction

The early Holocene witnessed gradual yet dramatic shifts in human lifeways, as early Neolithic human groups started to cultivate plants and domesticate animals (Harris 1996; Zeder 2008, 2017; Chessa et al. 2009, Arbuckle et al. 2014). Sheep, among the first such herded livestock species, were domesticated in southwest Asia c. 10,000 to 8,000 Before the Common Era (BCE) (Zeder 2008; Vigne 2011). Domestic sheep were eventually transported across the globe by humans, some becoming feral as in the case of the European Mouflon (Poplin 1979; Vigne et al. 2011; Barbato et al. 2017). Today, there exist hundreds of commercial domestic sheep breeds, multiple Mouflon lineages (i.e. wild relatives of domesticates, including feral sheep), and five other species of wild sheep (*Ovis*) worldwide. Despite intense work using genome data from modern-day sheep lineages (Lv et al. 2015, 2022; Barbato et al. 2017; Ciani et al. 2020; Deng et al. 2020; Li et al. 2020; Chen et al. 2021; Her et al. 2022; Wang et al. 2023), neither the location of, nor the wild progenitors involved in, the first domestication process, nor the post-Neolithic demographic histories of domestic sheep or Mouflon populations are well-understood.

One open question is the source of the domestic sheep gene pool. Earlier studies pointed out multiple Asiatic sheep species (*Ovis ammon*, *Ovis gmelini*, *Ovis vignei*) as the probable ancestor of domestic sheep (Nadler et al. 1973; Hiendleder et al. 1998; reviewed in Pedrosa et al. 2005). However, subsequent evidence has eliminated *O. ammon* and *O. vignei* as potential ancestors (Clutton-Brock 1981; Uerpmann 1987, Hiendleder et al. 1998). Some recent studies have considered the Iranian/Asiatic Mouflon (*O. gmelini*) from western Iran and easternmost Turkey as the wild population genetically closest to the ancestor of domestic sheep (reviewed in Pedrosa et al. 2005; Chen et al. 2021; Her et al. 2022; Cheng et al. 2023). However, this has not yet been fully established. The *O. gmelini* group has five subspecies: the Armenian Mouflon (*Ovis gmelini gmelini*), the Isfahan Mouflon (*Ovis gmelini isphananica*), the Laristan Mouflon (*O. gmelini laristanica*), the Cyprian Mouflon (*O. gmelini ophion*), and the Anatolian Mouflon (*O. gmelini anatolica*) (Blyth 1841). These groups are assumed to represent local wild populations since the early Holocene, except for the Cyprian Mouflon that dates back to the ∼12th millennium BCE and was presumably brought from mainland Anatolia/Levant (Zeder 2008; Vigne et al. 2011; Demirci et al. 2013; Sanna et al. 2015). Some genetic studies have also suggested that Anatolian and Cyprian Mouflons were subjected to proto/semi-domestication practices in the past (Hadjisterkotis 1992; Vigne 2003, 2011, 2014; Demirci et al. 2013; Sanna et al. 2015; Barbato et al. 2017). However, this is still a speculation, and the genetic relationships between these Mouflons and the ancient and present-day domestic sheep remain unclear.

Another largely unresolved question is the history of domestic sheep breeds (Chessa et al. 2009; Larson et al. 2014). Present-day domestic sheep cluster in two main geographic groups, Europe vs. Asia/Africa, based on genome-wide polymorphism data (Kijas et al. 2012; Naval-Sanchez et al. 2018; Li et al. 2020). This split is also observed in modern sheep mitochondrial haplotype groups, with European sheep mostly carrying haplotype B and Asian sheep dominantly carrying haplotype A (Bruford and Townsend 2006; Tapio et al. 2006; Meadows et al. 2007; Kijas et al. 2009; Demirci et al. 2013; Lv et al. 2015; Machová et al. 2022). The presence of an east–west genetic structure in modern breeds has previously been dated back to 7,000 to 6,000 BCE using molecular clock approaches (Niemi et al. 2013; Cai et al. 2018; Taylor et al. 2021). Accordingly, using ancient DNA, we recently showed that Anatolian Neolithic sheep (ANS) have a higher affinity to present-day European breeds than to non-European breeds, while Neolithic and Bronze Age Kyrgyzstan sheep show higher affinity to present-day Asian breed, suggesting the early establishment of this split (Yurtman et al. 2021). These patterns imply either multiple domestication centers and/or a large heterogeneous progenitor population that went through multiple independent bottlenecks. However, in the study by Yurtman et al. (2021), all modern breeds showed higher genetic affinity to each other than to Neolithic sheep. This could imply significant amounts of post-Neolithic admixture among continental sheep populations, including possible introgression from wild sheep into domestic flocks and the dispersal and breeding of sheep with desired traits across continents (Sherratt 1983; Marciniak 2011; Schoop 2014; Deng et al. 2020; Cheng et al. 2023). The latter scenario has already been explained using haplotype sharing information, with the most recent common ancestor of domestic breeds having been dated to c. 3,000 years ago (Ezard et al. 2009; Kijas et al. 2012).

**Fig. 1.**
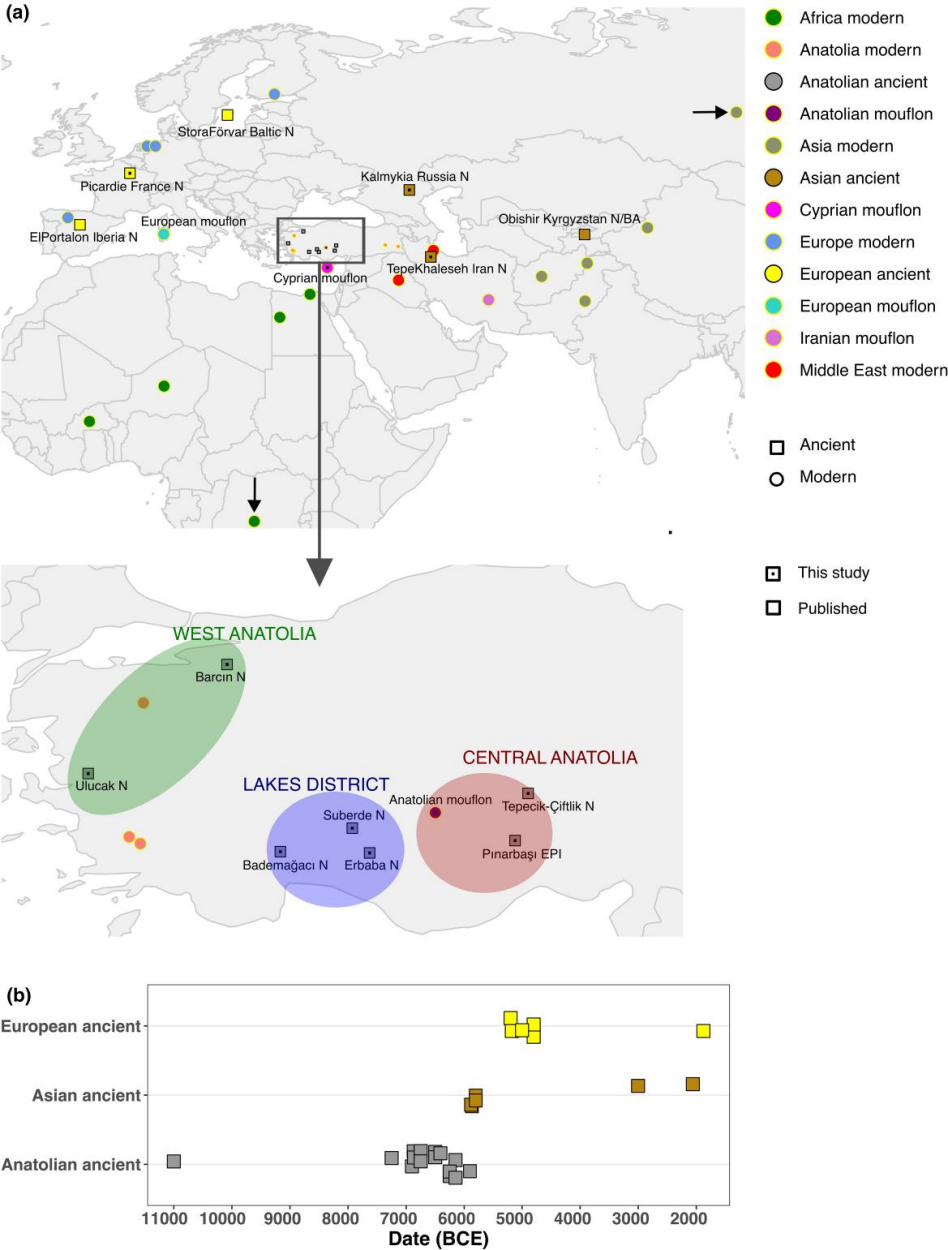
Map and timeline of all samples. a) The geographic origins of archeological and modern-day sheep samples studied in the present work and published sheep samples (supplementary table S1, Supplementary Material online). Subregions of Anatolia are depicted in the inset. West Anatolia (WA) is represented by Ulucak (6,750 to 6,150 BCE) and Barcın Höyük (∼6,250 BCE); the Lakes District (LD) includes Bademağacı (∼6,870 BCE), Suberde (∼7,250 BCE), and Erbaba (∼6,250 BCE). Central Anatolia is represented by Epipaleolithic Pınarbaşı (∼11,000 BCE) and Neolithic Tepecik-Çiftlik Höyük (∼6,900 to 5,900 BCE). b) Timeline of ancient samples grouped as European, Asian, and Anatolian.

Hence, paleogenomic data have already started providing clues into sheep demographic history (Taylor et al. 2021; Yurtman et al. 2021; Larsson et al. 2024). However, the limited spatial and temporal coverage, as well as the quality of the genetic data published, have impeded higher resolution analysis of sheep history, leaving questions as to the origin of domestication and the patterns of post-domestication dispersal open. To address this gap, here we present a comprehensive dataset of ancient and present-day wild and domestic sheep, including a 13,000-year-old Anatolian wild sheep genome, Eurasian domestic sheep paleogenomes, as well as the first genome-wide data from present-day Anatolian Mouflons (Table 1, Fig. 1). Our results identify Anatolian and Cyprian Mouflons and Epipaleolithic Anatolian sheep as better candidates for a domestication source than the Iranian Mouflon. We further confirm the presence of a dual structure of modern sheep diversity during the Neolithic using additional ancient genomes, including those from present-day Turkey and Iran. We also find multiple instances of population admixture or replacement, including post-Neolithic eastern influence on Baltic ancient sheep. Finally, as a legacy of domestication and recent bottlenecks, we see a depletion in the genetic diversities of present-day domestic and wild sheep.

**Table 1:** Archeological and genetic information of the ancient sheep

Sample ID	Date (cal BCE)	Location	Region	Genome coverage	Endogenous DNA	Genetic sex	mtDNA haplogroup	Number of SNPs
ASTF001	**2,007 to 1,749 BCE**	Stora Förvar	Baltic	0.976	0.109	XY	B1a	3,385,749
zah001	**2,125 to 1,995 BCE**	Zahanata	Kalmykia	0.020	0.021	XX	A1b, A1b_HM236175^a^	134,854
BFMC304A	4,900 to 4,700 BCE^b^	Picardie	France	0.965	0.103	XX	B1a, B1a2a^a^	3,745,289
BFMC304B	4,900 to 4,700 BCE^b^	Picardie	France	1.436	0.197	XX	B1a2a	4,091,273
MDVC564	5,100 to 4,900 BCE^b^	Picardie	France	0.794	0.165	XX	B1a2a1	3,497,447
APOR009	**5,310 to 5,042 BCE**	El Portalon	Iberia	1.986	0.103	XY	B1a1b, B1a2a1^a^	4,200,422
APOR008	**5,320 to 5,074 BCE**	El Portalon	Iberia	0.115	0.058	XX	B1a1, B1a1b, B1a2, B1a2a1^a^	779,239
tpc003	6,000 to 5,800 BCE^b^	Tepecik-Çiftlik	Anatolia	0.028	0.007	XX	B1a2a1	195,898
ira016	6,000 to 5,800 BCE^b^	Tepe Khaleseh	Iran	0.031	0.013	XX	na	221,913
ira011	6,000 to 5,800 BCE^b^	Tepe Khaleseh	Iran	0.031	0.014	XX	na	212,608
uhs031	**6,227 to 6,071 BCE**	Ulucak	Anatolia	0.056	0.028	XX	na	329,072
erb002	6,300 to 6,000 BCE^b^	Erbaba	Anatolia	0.0289	0.0092	XX	na	216,540
bar001	6,300 to 6,200 BCE^b^	Barcın	Anatolia	0.0229	0.0074	XX	na	148,823
tps083	**6,469 to 6,361 BCE**	Tepecik-Çiftlik	Anatolia	0.0471	0.0117	XY	A1b, A1b_HM236175^a^	364,410
ERB455	6,500 to 6,000 BCE^b^	Erbaba	Anatolia	0.0080	0.0277	XY	B1a1b	59,946
TPC039	6,600 to 6,400 BCE^b^	Tepecik-Çiftlik	Anatolia	0.0203	0.0608	XX	D, D_HM236181^a,c^	153,633
TPC037	6,600 to 6,400 BCE^b^	Tepecik-Çiftlik	Anatolia	0.0627	0.1184	…	B1a1b	454,792
ulu010	7,000 to 6,500 BCE^b^	Ulucak	Anatolia	0.0086	0.0047	XX	B1a1b	54,892
ulu012	7,000 to 6,500 BCE^b^	Ulucak	Anatolia	0.0432	0.0127	XX	B1a1b	260,722
tps062	**7,031 to 6,687 BCE**	Tepecik-Çiftlik	Anatolia	2.3050	0.1245	XX	B1a2a	4,210,271
bad003	7,035 to 6,705 BCE^b^	Bademağacı	Anatolia	0.0206	0.0068	XX	na	133,242
tps001	**7,059 to 6,756 BCE**	Tepecik-Çiftlik	Anatolia	0.1531	0.0233	XX	B, B1a1b	1,009,687
sub008	7,500 to 7,000 BCE^b^	Suberde	Anatolia	0.0109	0.0051	XY	na	73,432
pbh003	11,500 to 11,000 BCE^b^	Pınarbaşı	Anatolia	0.0514	0.0045	XX	B1a1b	398,235

^a^More than one entry means that there is some uncertainty in the haplogroup inference, with variants missing and/or present in both of the options listed.

^b^Date intervals based on archeological context (i.e. relative dating), shown in standard font, or calibrated radiocarbon dates, shown in bold.

^c^We used a relaxed filtering setup to assemble this mitogenome due to low data quality.

## Results and Discussion

### Sampling and Genomic Data Production

We screened *n* = 238 ancient putative sheep skeletal samples spanning late Pleistocene and early Holocene Eurasia to the present day (Table 1, supplementary table S1, Supplementary Material online) from Anatolia, Kalmykia, and Iran. We could identify only 18 samples with moderate to low endogenous DNA content (between 0.4% and 12%, median = 1.2%) (Table 1, supplementary table S1, Supplementary Material online), a low proportion possibly due to food processing as well as petrous bone and tooth tissue being unavailable (Supplementary Note S1, Supplementary Material online). These 18 samples were confirmed as sheep (*Ovis*) using the MTaxi algorithm (Atağ et al. 2022) and shotgun sequenced further. We also joined the resulting data with two unpublished Mid-Holocene domestic sheep genomes from Iberia and one from the Baltic Sea, produced as part of parallel studies (Morell Miranda 2023; Larsson et al. 2024), together with three unpublished Neolithic sheep genomes from France (Table 1, supplementary table S1, Supplementary Material online). Our combined ancient dataset included 24 new ancient genomes with autosomal coverage of 0.008 to 2.3× (median 0.045×) (Table 1, supplementary table S1, Supplementary Material online). All paleogenome data exhibited patterns of postmortem DNA damage (22% to 50% at 5′-ends) and relatively short average read lengths (mean/median = 70 bp, between 54 and 103 bp), as expected from authentic ancient DNA (supplementary table S1, Supplementary Material online). Except for a single Epipaleolithic Anatolian sample from Pınarbaşı, all ancient individuals were derived from assemblages identified as largely domestic by zooarchaeologists (supplementary Note S2, Supplementary Material online). In addition to ancient sheep, we shotgun sequenced present-day Anatolian Mouflons (*n* = 5), and Cyprian Mouflons (*n* = 2), to coverages 0.42× to 16.9× (median 1.47×) (supplementary table S1, Supplementary Material online). We merged these data with selected published present-day Eurasian and African modern-day breeds (Naval-Sanchez et al. 2018; Deng et al. 2020; Li et al. 2020) as well as Neolithic and Early Bronze Age (EBA) (7,012 to 3,003 BCE) Kyrgyzstan sheep (Obishir V) (Taylor et al. 2021) (supplementary table S2, Supplementary Material online).

In downstream analyses, we used two alternative approaches. First, to minimize SNP ascertainment bias toward modern commercial breeds (Wang and Nielsen 2012), we created an outgroup-ascertained c. 4.6 million SNP panel by de novo SNP calling with modern-day wild sheep species (*Ovis ammon*, *Ovis ammon polii*, *Ovis canadensis*, *Ovis dalli*, *Ovis nivicola*) expected to be outgroups to domestic sheep and their close wild relatives (Chen et al. 2021) (Materials and Methods) (supplementary table S3, Supplementary Material online). Second, we calculated *f-*statistics using de novo variants identified in each ancient genome, excluding transitions.

### The Anatolian Epipaleolithic Sheep Genome Shows a Stronger Affinity to Domesticates Than the Iranian Mouflon

**Fig. 2.**
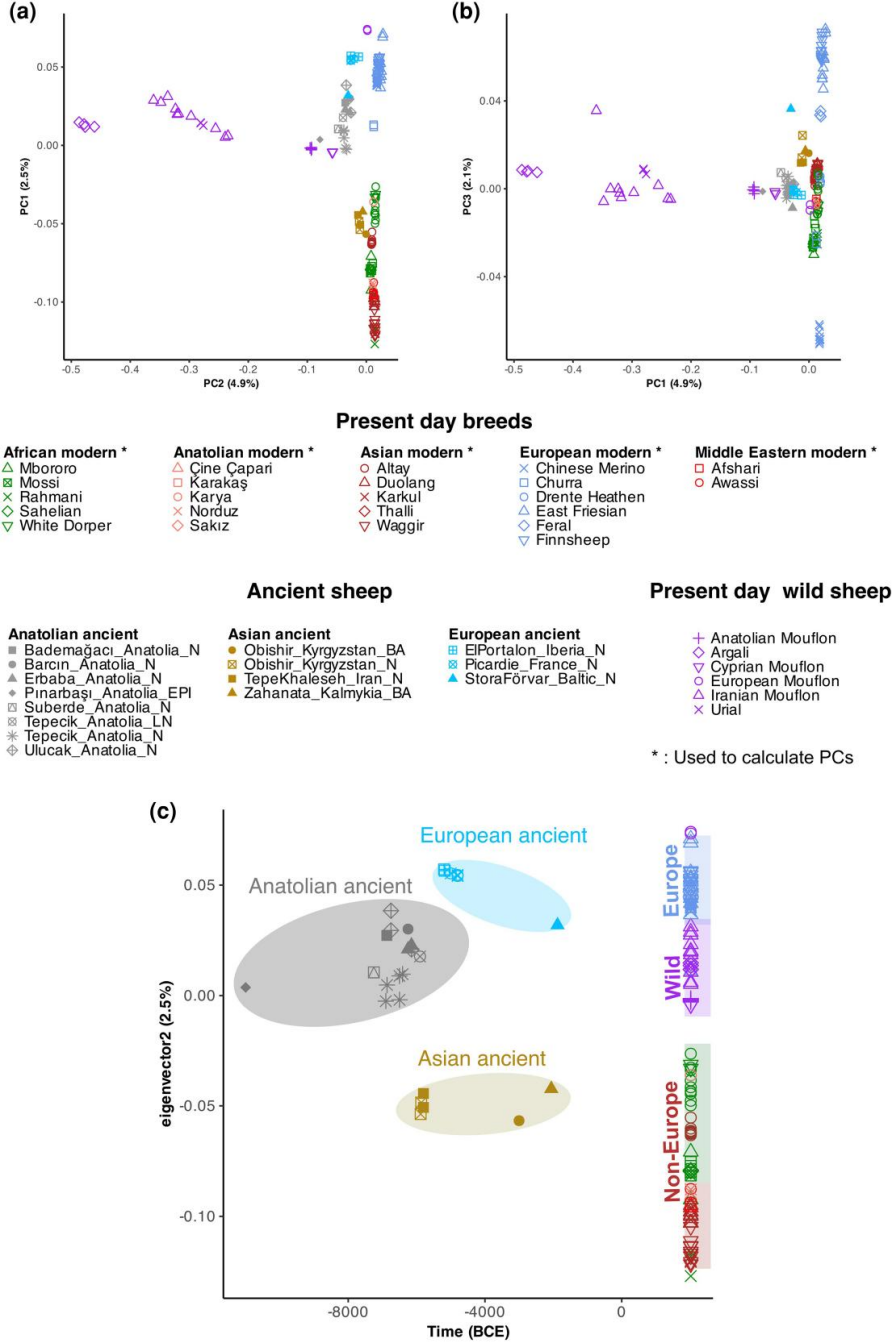
Principal component analysis (PCA) of genome-wide diversity. PCA plot describing the genetic affinities among ancient and modern populations studied. The genotype of each ancient individual was projected upon the first two PCs calculated using 39 present-day sheep breeds (indicated with asterisks in the key, and in supplementary table S2, Supplementary Material online) using the SNP panel. The percentages on the *x* and *y* axes show the proportion of variance explained by a) PC1 and PC2, respectively. b) PC1–PC3. PCA plot with PC2–PC3 is shown in supplementary fig. S3, Supplementary Material online. c) PC2 values plotted against sample dates (years BCE).

We first summarized genome-wide affinity patterns among ancient and present-day sheep lineages through principal component analysis (PCA) (Fig. 2) using the outgroup-ascertained SNP dataset. We projected the 27 ancient (including three published from Kyrgyzstan) and 7 newly generated Mouflon genomes onto the first two principal components calculated from the genetic diversity of published modern-day breeds and Asian and European Mouflons (supplementary table S3, Supplementary Material online). PC1 reflects the differentiation between wild and domestic groups (4.8% of the variation), while PC2 separates domestic sheep into European and non-European clusters (2.6% of the variation). Argali (*O. ammon*), Urial (*O. vignei*), and Iranian Mouflon (*O. gmelini*) were the most distant wild sheep genomes to the modern and ancient domestic sheep in this PC space, while the present-day Anatolian Mouflon and the Cyprian Mouflon, as well as the Anatolian Epipaleolithic sheep were closer to domestic sheep. The European Mouflon fell inside domestic diversity as expected from its feral status. The same PCA without the Argali group can be seen in supplementary fig. S1, Supplementary Material online. General pattern observed was further confirmed with PCA constructed with 50 K SNP data (Kijas et al. 2012) (supplementary fig. S2, Supplementary Material online).

**Fig. 3.**
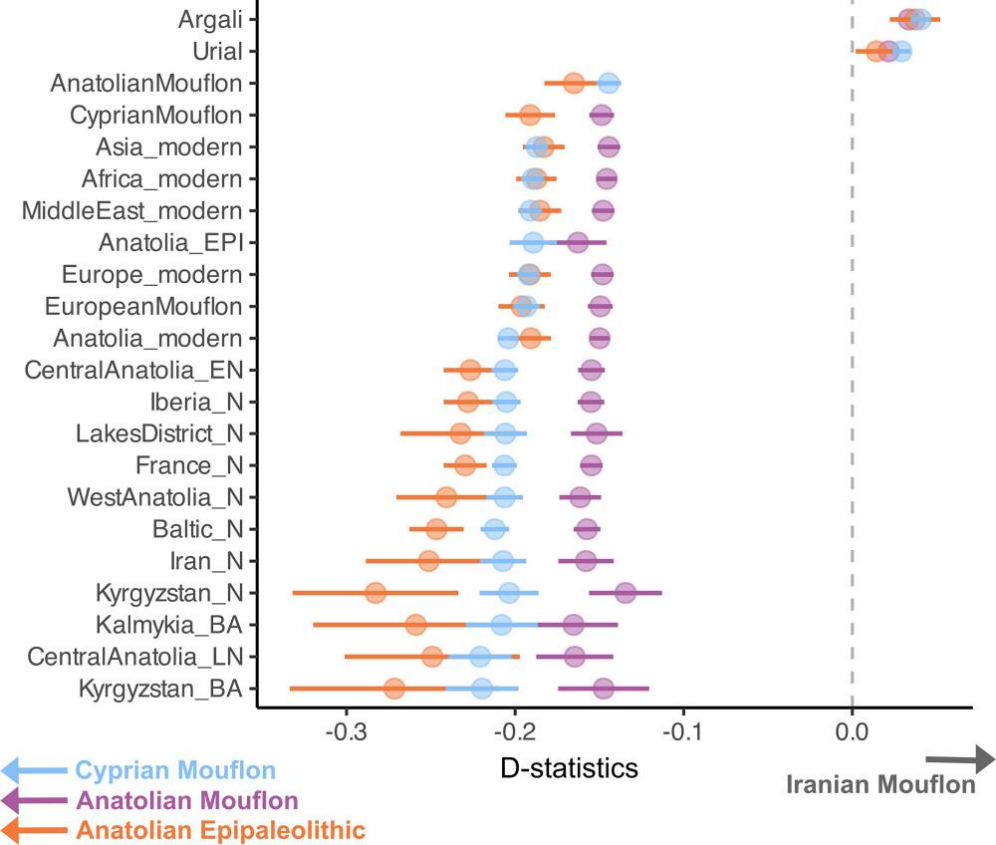
*D*-statistics calculated as *D(Goat, Y; Anatolian Mouflon/Cyprian Mouflon/Anatolian Epipaleolithic, Iranian Mouflon)*. Negative values indicate higher genetic affinity between *Y*, i.e. the sheep populations shown on the *y* axis, and the Anatolian Mouflon/Cyprian Mouflon/Anatolian Epipalaeolithic, relative to the Iranian Mouflon. *D*-statistics were calculated with angsd (Korneliussen et al. 2014) using de novo variants. Error bars show ±3 standard errors. All tests were nominally significant (100% |Z| > 3, no multiple testing correction applied).

The Iranian Mouflon is frequently assumed to be the closest wild relative of domestic sheep (Zeder 2008; Her et al. 2022; Cheng et al. 2023). However, in the PCA, both the Anatolian Epipaleolithic sheep, as well as the Anatolian Mouflon and Cyprian Mouflon, were closer to domestic sheep than was the Iranian Mouflon. To confirm this, we calculated *D*-statistics of the form *D(Goat, X; Anatolian Mouflon/Cyprian Mouflon/Anatolian Epipaleolithic, Iranian Mouflon*), where *X* is any ancient or present-day domestic sheep, using de novo-called variants in each comparison (Materials and Methods). *D*-statistics involving both ancient and present-day domestic lineages were all significantly negative (100% of 57 tests |Z| > 3, no multiple testing correction applied) (supplementary table S4, Supplementary Material online), indicating that domestic sheep are genetically closer to Anatolian Epipaleolithic, Anatolian Mouflon, and Cyprian Mouflon than the Iranian Mouflon (Fig. 3). This is interesting as Anatolian Epipaleolithic sheep derive from Central Anatolia, where early sheep management is well documented (Stiner et al. 2022). Meanwhile, sheep were introduced to Cyprus by 8,000 BCE (Vigne et al. 2011), likely from mainland Anatolia. We note that Neolithic human populations in Cyprus were also found to be closely related to contemporaneous Central Anatolians (Lazaridis et al. 2022).

These observations tentatively suggest that sheep domestication occurred on the Anatolian plateau, to the northwest of the conventionally assumed borders of the Fertile Crescent, rather than inside the Eastern Fertile Crescent, Zagros/Iran. This would also be compatible with a recent study showing a lack of shared mitochondrial haplotypes and only one shared Y-chromosome haplotype between Iranian Mouflon and domestic sheep (Wang et al. 2023). However, the evidence is yet partial. The history of uniparental markers may not represent the average history of a lineage. Moreover, the genetic distance between Iranian Mouflon and domestic lineages relative to Anatolian Epipaleolithic, Anatolian Mouflon, and Cyprian Mouflon could be related to post-domestication gene flow into Iranian Mouflon from a more distinct wild lineage. In fact, this is not unlikely as we find *D(Goat, Urial; Anatolian Mouflon/Cyprian Mouflon/Anatolian Epipaleolithic, Iranian Mouflon)* as well as *D(Goat, Anatolian Mouflon/Cyprian Mouflon/Anatolian Epipaleolithic; Urial, Iranian Mouflon)* significantly positive (100% of three tests |Z| > 3 for both, no multiple testing correction applied) (supplementary table S4, Supplementary Material online, supplementary table S5, Supplementary Material online) (also noted by Chen et al. 2021). We hence cannot yet rule out that Zagros or North Mesopotamian wild sheep populations were another domestication source.

### Early Diversification of Domestic Sheep in Southwest Asia

The PCA reveals two distinct clusters of modern-day domestic sheep: European and non-European (Asian and African) breeds (Fig. 2). ANS, comprising domestic sheep genomes from six settlements and spanning ∼7,250 to 5,900 BCE, were closer to the European cluster in PC space. The Iranian Neolithic sheep (∼7,000 to 6,000 BCE), Kyrgyzstan Neolithic/Bronze Age, and Kalmykia Iron Age sheep were all closer to the non-European cluster (Fig. 2).

**Fig. 4.**
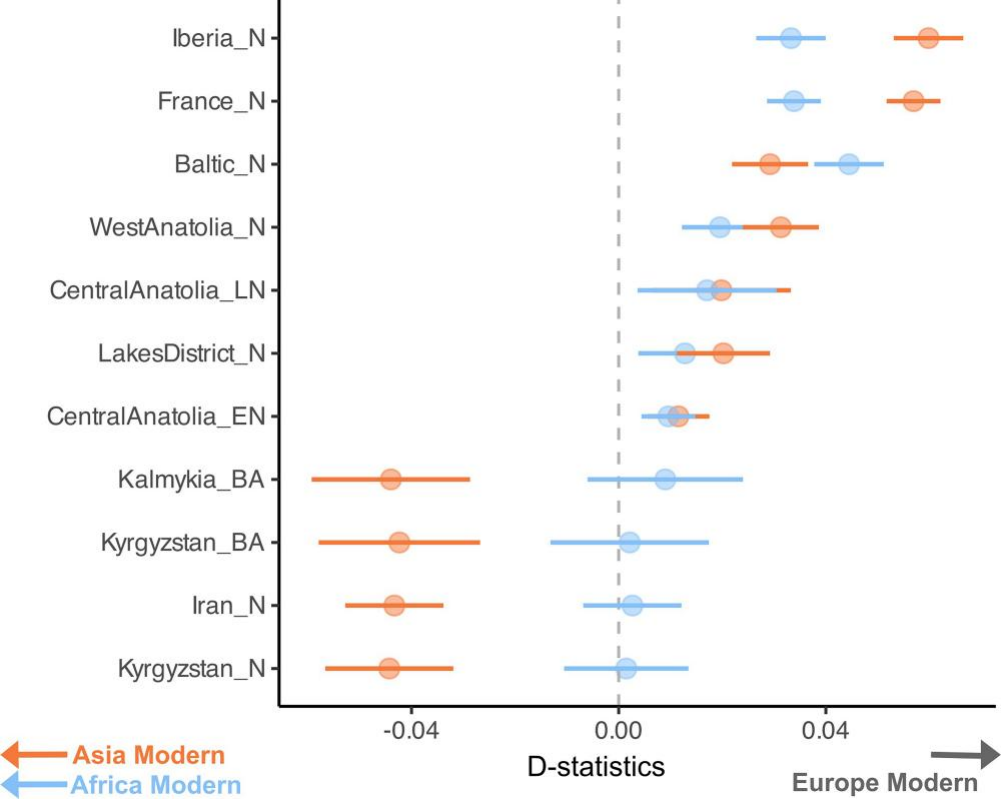
*D*-statistics of the form *D(Goat, Anatolian/Asian/European ancient; non-EU, EU)*. EU, European modern-day breeds; non-EU, Asian and African modern-day breeds. The orange points show comparisons between modern-day European and Asian breeds, and the blue points show comparisons between modern-day European and African breeds. Positive values indicate ancient genomes showing higher affinity to EU, while negative values indicate higher affinity to non-EU. *D*-statistics were calculated with angsd (Korneliussen et al. 2014) using de novo variants. Error bars show ±3 standard errors (no multiple testing correction applied).

To study this in more detail, we separated ANS into four spatiotemporal groups: Lakes District, West Anatolia, Central Anatolia Early, and Central Anatolia Late (Fig. 1a). The latter two groups were based on the observation that the five genomes from early Neolithic Tepecik-Çiftlik Höyük (6,900 to 6,410 BCE) were occupied a distinct location on the PCA (Fig. 2, Tepecik_Anatolia_N) relative to the single ∼5,900 BCE individual (tpc003) from the same site (Fig. 2, Tepecik_Anatolia_LN). We then compared ancient Anatolian and Asian lineages with modern breeds using D-tests. Consistent with PCA results, we found that different ANS groups all showed higher affinity to European over non-European modern breeds (100% of eight tests of the form *D(Goat, ANS; EU, non-EU)* with |Z| > 3, no multiple testing correction applied, supplementary table S6, Supplementary Material online), while, Asian ancient sheep (Iran N, Kyrgyzstan N and BA, Kalmykia BA) tended to show affinity to Asian modern breeds (|Z| > 3 in 100% of eight tests, no multiple testing correction applied, supplementary table S6, Supplementary Material online) (Fig. 4). Both results agree with our previous observation of the east–west diversification of domestic sheep within SW Asia as early as the 7th millennium BCE (Yurtman et al. 2021).

**Fig. 5.**
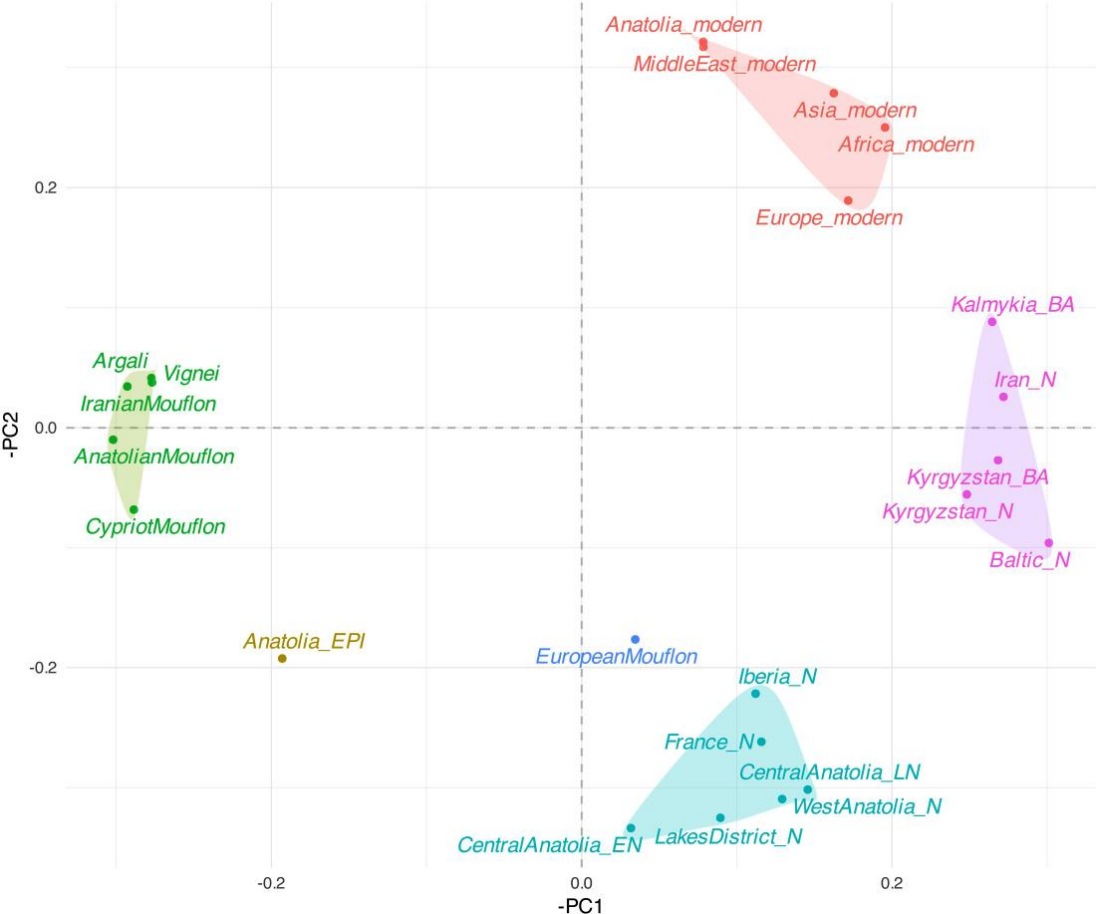
*D*-PCA summarizing *D*-statistics across all quadruple combinations of sheep populations. We calculated *D*-PCA using *D*-statistics of the form *D(A; B, C, D)*, where A to D are any ancient or present-day sheep populations. *D*-statistics were calculated with angsd (Korneliussen et al. 2014) using de novo variants. We then performed a PCA on these data (Materials and Methods).

We next performed a *D*-PCA on ancient and modern sheep populations for studying genetic clustering; this method has the advantage of not being constrained by diversity among modern-day lineages (Bergström et al. 2020) (Fig. 5, supplementary table S7, Supplementary Material online). This revealed four clusters: one that included wild sheep and Mouflons (except for the European Mouflon), a second with all present-day domestic breeds, a third that included all Anatolian, French, and Iberian Neolithic genomes, and a fourth comprising Asian and Baltic Neolithic and Bronze Age genomes. The Anatolian Epipaleolithic and European Mouflon genomes were located between the Anatolian Neolithic cluster and the cluster including other wild sheep. The central positioning of Anatolian Epipaleolithic sheep among the domestic and wild populations hints at the Central Anatolian wild populations being the ancestors of domestic sheep.

These observations could be compatible with a scenario with two domestication centers, one in Anatolia, and another in North Mesopotamia/Zagros, involving differentiated gene pools. Under this scenario, we might expect a clear population structure among Neolithic sheep. We tested this in various ways. *D*-statistics of the form *D(Goat, ANS; ANS, Iran_Neolithic)* revealed no clear differentiation between ANS and Iran Neolithic (100% of 12 comparisons |Z| < 3, no multiple testing correction applied, supplementary table S8, Supplementary Material online, supplementary fig. S4, Supplementary Material online). We also found that Anatolian Epipalaeolithic were symmetrically related to ANS and Iran Neolithic sheep, as were more recent ancient sheep (in 21 of 28 [75%] comparisons (|Z| < 3, no multiple testing correction applied, supplementary table S8, Supplementary Material online, supplementary fig. S4, Supplementary Material online). Finally, testing *D(Goat, ANS; ANS, ANS)* (supplementary table S9, Supplementary Material online, supplementary fig. S5, Supplementary Material online) revealed no major clustering among spatiotemporal groups in Anatolia. Thus, despite clustering patterns emerging in the PCA and *D*-PCA, direct D-tests do not reveal strong population structure in the Neolithic-SW Asia sheep gene pool.

This lack of structure could be compatible with two scenarios. First, two progenitor sources may have been involved in domestication in the east and west of SW Asia including outside the Fertile Crescent, but these may have not been highly differentiated from each other. Alternatively, sheep domestication may have involved a single wild gene pool and the observed east–west differentiation may have been caused by post-domestication drift, or introgression from different wild populations.

The aDNA evidence presented herein for domestication of local wild sheep in central Anatolia is highly congruent with independent archeological evidence for the early development of sheep herding in this region. Both in the Konya plain, the sub-region wherein aDNA of Pınarbaşı wild sheep is documented, at the site of Boncuklu (Baird et al. 2018) and in Cappadocia at Aşıklı (Stiner et al. 2022), there is early evidence for sheep management/herding between c. 8,300 and 8,100 cal BCE. This suggests that these practices were widespread across central Anatolia in the second half of the 9th millennium cal BCE. The evidence is provided by multiple types of proxies. At both of these sites, herbivore dung is found on site, as fuel at Boncuklu (Garcia-Suarez et al. 2020; Portillo et al. 2020). At Aşıklı, it is present on site with chemical (high levels of urine salts) and micromorphological evidence of penning and, in addition, high levels of perinatal and neonatal mortality suggesting that aborting and birthing sheep were present on site (Stiner et al. 2022) indicative of direct management. At Boncuklu, isotope evidence indicates elevated δ15N compared to earlier wild sheep including Epipalaeolithic and 10th to 9th millennium sheep from Pınarbaşı (Middleton 2018). This last case probably indicates translocation of caprines from surrounding hills to the more arid plain in the center of the Konya basin.

This evidence from central Anatolia is as early as any other convincing indicators for caprine management elsewhere in SW Asia suggesting local central Anatolia processes leading to herding and domestication were occurring in the 9th millennium cal BCE. Synchronously, there is evidence for sheep management in SE Anatolia. For example, at Nevalı Çori, Peters et al. (2005) make a convincing case for sheep management through culling profiles combined with evidence of some smaller sized animals in the second half of the 9th millennium cal BCE and Lösch et al. (2006) suggest that isotopes indicate foddering of these smaller sized sheep. Similar evidence is documented at Çayönü (Hongo et al. 2009). In addition, sheep have been translocated to Cyprus by c. 8,300 to 8,000 cal BCE at Kissonerga Mylouthkia and Shillourokambois (Vigne et al. 2011), as the aDNA evidence in this paper suggests probably from the Anatolian plateau via the southern coastline of Anatolia, indicating at least certain levels of management.

The transformation of managed sheep in central Anatolia into morphologically evidenced domesticates is suggested by c. 7,500 cal BCE, where at Canhasan III, increased frequencies of caprines (relative to earlier Boncuklu) are seen with many sheep showing elevated δ15N, the same isotope signature as seen at Boncuklu, combined with C4 plants in the diet, suggesting management reflected in changing diets of the sheep (Middleton 2018). Likewise, at Aşıklı, it is suggested that the herded sheep of the 9th millennium become domesticated during the 8th millennium cal BCE (Stiner et al. 2022). This trend of increasing importance of the local domestic sheep is further indicated by the evidence from Çatalhöyük where morphologically domestic caprines, largely sheep, dominate faunal assemblages in terms of frequency, from the late aceramic Neolithic c. 7,100 cal BCE.

### The Impact of Neolithic Dispersals into Central Asia and Europe on Genetic Diversity

Starting with the 7th millennium BCE, domestic sheep were transported by Neolithic people from SW Asia to the northeast into the Caucasus (Chataigner et al. 2014), to the east into Central Asia (Taylor et al. 2021), and to the west into Europe (Price 2000; Colledge et al. 2005; Harris and Gosden 2010; Arbuckle et al. 2014; Lv et al. 2015). The Kyrgyz and Iberian/French Neolithic genomes in our dataset represent these latter two dispersal events. The clustering of Kyrgyz sheep with those from Iran and of Iberian/French sheep with those from Anatolia (Fig. 5), even if not significant in individual D-tests (supplementary fig. S4, Supplementary Material online, supplementary table S8, Supplementary Material online), implies that these were derived from the eastern- and western-most domestic sheep populations of the Neolithic-SW Asia.

**Fig. 6.**
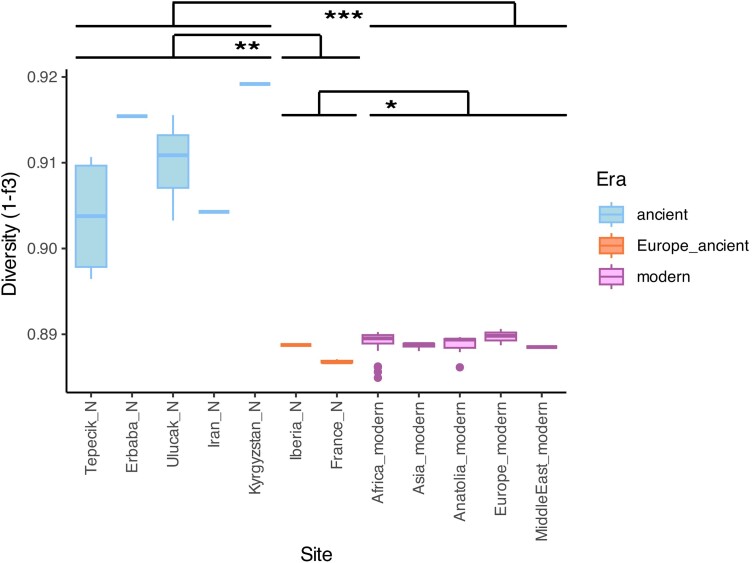
Within-population genetic diversities of ancient and modern-day domestic sheep. Diversities were calculated using pairwise (1 − outgroup *f*_3_) between genomes from each site/breed as a distance measure, shown on the *y* axis. The *x* axis depicts archeological sites for ancient sheep populations (left) or regions of origin for modern-day sheep breeds (right). The pairwise 1 − *f*_3_ statistics were calculated with the outgroup-ascertained SNP panel using only transversions. The boxplots show diversity measurements for sites with more than two genomes, while single lines indicate estimates for sites that include only two genomes. The 1 − *f*_3_ values were used to compare diversities of Anatolia/Asia ancient, Europe ancient, and modern groups with the Kruskal–Wallis rank sum test (*P* = 5e^−08^). Between-group comparisons were further tested using the Wilcoxon rank sum test (*P*_(Anatolia/Asia ancient-modern)_ = 8e^−08^, *P*_(Anatolia/Asia ancient-Europe ancient)_ = 0.0011, *P*_(Europe ancient-modern)_ = 0.014).

We hypothesized that founder effects during human-mediated dispersal might have eroded diversity in sheep populations. To test this, we determined within-site pairwise genetic distances in archeological sites represented with ≥2 genomes and calculating distances as (1 − outgroup *f*_3_), using SNPs restricted to transversions (Fig. 6, supplementary table S10, Supplementary Material online) or using all SNPs (supplementary fig. S6, Supplementary Material online, supplementary table S11, Supplementary Material online). This revealed relatively high diversity levels across Neolithic-SW Asia, including Iran, and also high values for the pair of Kyrgyzstan Neolithic genomes. In contrast, all modern-day breeds have significantly lower diversity than the Anatolian and Asian ancient groups analyzed (Kruskal–Wallis rank sum test, *P* = 3e^−10^).

Intriguingly, and in contrast to the Kyrgyz sample, the Iberian and northern French Neolithic genomes also harbored lower diversity compared to the Anatolian and Iranian Neolithic sheep genomes. This diversity is lower than any other pair of ancient sheep genomes in our dataset and on a par with modern-day breeds (Fig. 6, supplementary table S10, Supplementary Material online). This observation implies differences in the sheep transport dynamics between the European and Central Asian land routes, with only the former involving a strong bottleneck. Two routes of dispersal have been hypothesized to extend from Anatolia to Europe, the continental route and the Mediterranean maritime route (Shennan 2018; Racimo et al. 2020; Brigand et al. 2022). The northern French Neolithic sheep arrived in the Paris Basin with the Linear Pottery Culture (LBK), known to have spread west from the middle Danube into the Rhine and Seine basins, as well as north and east into the Elbe and Vistula basins (Arbogast 1994; Hachem 2011, 2018; Auxiette and Hachem 2021). Iberian Neolithic sheep were potentially introduced through the Mediterranean maritime route considering the location and date of the settlement. The same low diversity pattern observed in both French and Iberian genomes either indicates an earlier bottleneck occurring in Southeastern Europe, after the dispersal of sheep from Anatolia over the land route; or both French and Iberian sheep may be derived from the maritime route, which is highly unlikely since the French Neolithic sheep belong to a continental Early Neolithic context (LBK and Blicquy/Villeneuve-Saint-Germain). A third and least parsimonious scenario may be that Iberian and French sheep spread through distinct routes (land and maritime, respectively), while in parallel undergoing independently similar bottlenecks. We note that the Mediterranean bottleneck and resulting low genetic diversity observed in sheep is paralleled by a similar finding in the human population of Neolithic Iberia that was also characterized by a lower genetic diversity relative to Neolithic Anatolia and Central Europe (Valdiosera et al. 2018).

### Evidence for an Early Admixture/Replacement Event in Central Anatolia

A number of observations from the PCA and *D*-PCA pointed toward admixture and/or replacement events in the history of domestic sheep. One such observation was the difference in genetic profile between Central Anatolia Early and Central Anatolia Late Neolithic sheep, derived from the same site (Tepecik-Çiftlik) but separated by ∼500 years (Figs. 2 and 5). In fact, the Central Anatolia Early genomes from the mid-7th millennium layers of Tepecik-Çiftlik were more distinct from all the other Neolithic Anatolian groups. Most notably, the West Anatolia sheep sample showed strong affinity toward Central Anatolia Late in the PCA and *D*-PCA, a pattern confirmed by testing *D(Goat, X; Central Anatolia Late, Central Anatolia Early)* (16 of 21 [76%] tests |Z| < 3, without correction for multiple testing) (supplementary table S12, Supplementary Material online, supplementary fig. S7, Supplementary Material online). This pattern may imply that Central Anatolia Early was an early domestic assembly that was partly replaced or became highly admixed by the early 6th millennium BCE. This introgression could presumably be from sheep populations from further East, such as Upper Mesopotamia, that also influenced West Anatolia. This may be expected given the level of inter-regional mobility among Neolithic human groups inferred from aDNA (Altınışık et al. 2022; Lazaridis et al. 2022).

### Eastern Influence Remodeled the European Sheep Gene Pool by the Bronze Age

**Fig. 7.**
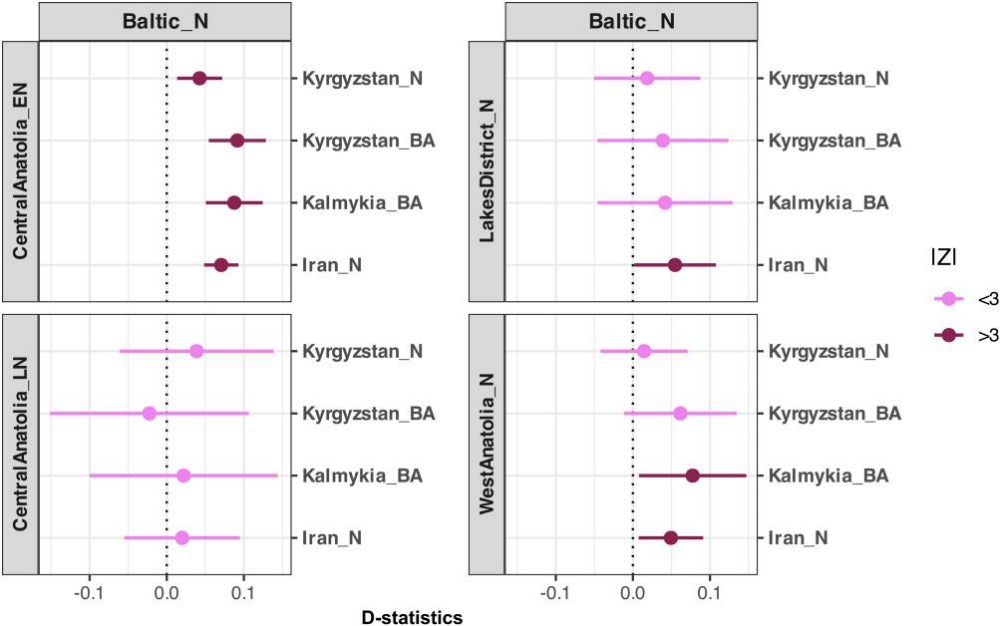
*D*-statistics indicate admixture in Baltic Neolithic sheep. The graph shows *D*-statistics of the form *D(Goat, Baltic_N; ANS, Asia ancient)*, calculated with angsd (Korneliussen et al. 2014) using de novo variants. Error bars show ±3 standard errors (no multiple testing correction applied).

Another surprising observation from the *D*-PCA relates to the Baltic Neolithic sheep genome of the 2nd millennium BCE, a period that corresponds to the Bronze Age in many regions of Eurasia. Although this genome clustered with ancient Anatolian genomes in the PCA (Fig. 2) and has a higher affinity to modern-day European breeds over Asian breeds (Fig. 4), in the *D*-PCA, it clustered with ancient Asian genomes from Iran, Kyrgyzstan, and Kalmykia (Fig. 5). The latter pattern was further supported by significantly positive results for *D(Goat, Baltic Neolithic; ANS, Asia Ancient)* (7 of 16 [44%] tests |Z| > 3, without correction for multiple testing) (Fig. 7, supplementary fig. S1, Supplementary Material online, supplementary table S13, Supplementary Material online). Meanwhile, we also find that ANS and Iberia Neolithic sheep genomes were genetically closer to Baltic Neolithic than to ancient Asian sheep (supplementary fig. S8, Supplementary Material online, supplementary table S14, Supplementary Material online). This suggests that Baltic Late-Neolithic sheep may be the result of admixture between an early population of possibly Anatolian-related sheep, and incoming Asian sheep. Using *f*4-ratio analysis with the model *f4(Iran_N, Goat; Baltic_N, Iberia_N/France_N) / f4(Iran_N, Goat; Kyrgyzstan_N, Iberia_N/France_N)*, we estimated the Baltic Late-Neolithic genome to carry ∼70% admixture from a Kyrgyzstan Neolithic-related source relative to a European Neolithic sheep background (supplementary table S15, Supplementary Material online) (see Materials and Methods). In a parallel study, the same Baltic Neolithic genome used here (ASTF001) was modeled as a mixture of 88% from a source related to northern European sheep and 12% from an unobserved population basal to all other domestic sheep in an admixture graph analysis (Larsson et al. 2024). These observations indicate an Asian influx into north European sheep breeds by the Bronze Age.

We then asked whether such influx may have left a permanent signature in the European sheep gene pool. Supporting this notion, we found that modern European breeds show higher affinity toward Iranian Neolithic than to ANS in D-tests (two of four [50%] tests with |Z| > 3, without correction for multiple testing), in contrast to the PCA (supplementary fig. S1, Supplementary Material online, supplementary table S8, Supplementary Material online). This suggests that Anatolian ancestry in European sheep diminished in time via Asia-related admixture, possibly during the Bronze Age, as represented by the Baltic genome. Importantly, this involved not only northern but also southern European breeds, indicating that the effect spread through the continent (61 of 96 [64%] tests with |Z| > 3, without correction for multiple testing, supplementary table S16, Supplementary Material online). Such admixture would also explain why modern-day European breeds tend to have higher within-population diversity levels than sheep from Neolithic France or Iberia (Fig. 6), despite intense breeding in the modern period.

It is tempting to speculate that this admixture we identify in sheep may be related to the 3rd-millennium Pontic Steppe (Yamnaya) ancestry expansion (Sherratt 1981) and admixture in humans across Europe (Allentoft et al. 2015; Haak et al. 2015). Notably, it has been hypothesized that the Pontic Steppe expansion was linked to dairying in addition to horse domestication (Wilkin et al. 2021; Scott et al. 2022), or that it may be related to the secondary product revolution in sheep that includes specific wool sheep dispersal (Vigne and Helmer 2007; Greenfield 2010). Irrespective of the driving force, however, we are likely observing both people and livestock from the Pontic Steppe moving into Europe and admixing in large numbers during the Bronze Age, bringing more eastern genomes and diluting the more local Anatolian Neolithic-related genomes.

### Indication of Recent Admixture Shaping Modern-Day Breeds

We next asked whether the domestic sheep gene pool remained stable after the Bronze Age. Indeed, we find evidence for regional continuity, such as present-day Asian breeds being closer to Iran Neolithic or Kyrgyzstan Bronze Age sheep than to European present-day breeds, and likewise, present-day European breeds being closer to the Baltic Neolithic genome than to all other present-day breeds (100% of four tests with |Z| > 3, without correction for multiple testing) (supplementary table S17, Supplementary Material online). However, we also noticed patterns suggesting further admixture that may have shaped the domestic sheep pool. The first was the unexpected clustering of all modern-day genomes in the *D*-PCA, separate from either ancient Anatolian-related or Asian-related clusters (Fig. 5). Second, we noticed that Neolithic sheep from Anatolia, France, and Iberia were all closer to Iran Neolithic, Kyrgyzstan Neolithic, Kalmykia Bronze Age, or Kyrgyzstan Bronze Age than modern-day genomes from Asia, the Middle East, or Africa (71 of 72 [99%] tests with |Z| > 3, without correction for multiple testing, supplementary table S17, Supplementary Material online). The Iranian Neolithic genomes were also closer to Kyrgyzstan Bronze Age sheep compared to any modern-day breed (supplementary table S17, Supplementary Material online). These observations raise the possibility of gene flow into Asia from unobserved populations, possibly post-Bronze Age, that diverged the modern-day gene pool.

Another intriguing pattern involved ancient sheep choosing Anatolian Epipaleolithic over Cyprian Mouflon (all 11 comparisons |Z| < 0 and 4 with |Z| < −3, without correction for multiple testing) when testing *D(Goat, Ancient; Anatolian Epipaleolithic, Cyprian Mouflon)* (supplementary fig. S9, Supplementary Material online, supplementary table S18, Supplementary Material online), while all five present-day continental populations choose Cyprian Mouflon when testing *D(Goat, X; Anatolian Epipaleolithic, Cyprian Mouflon)* (100% of five tests with |Z| > 3, without correction for multiple testing, supplementary table S18, Supplementary Material online). This was unexpected because both Anatolian Epipaleolithic and Cyprian Mouflon might be considered outgroups to all domestic sheep. We could rule out this being a technical artifact related to attraction between modern-day vs. paleogenomes, as modern-day Mouflons did not choose Cyprian Mouflon in the same test. This has two implications. First, it suggests that Cyprian Mouflon received domestic gene flow in the recent past (Atağ et al. 2024). Accordingly, Cyprian Mouflon shows asymmetric affinity to Anatolian present-day sheep over Asian, European, or African breeds (100% of four tests with |Z| > 3, without correction for multiple testing, supplementary fig. S10, Supplementary Material online, supplementary table S19, Supplementary Material online). Second, it indicates that all modern breeds share some ancestry not represented by the ancient genomes in our dataset (up to the 2nd millennium BCE) but present in Cyprian Mouflon.

The evidence for unique ancestry in modern-day breeds and extra affinity toward Asia together could be explained by an introgression event, possibly in Asia, and the subsequent spread of that ancestry throughout sheep breeds within the last two millennia. This could originate from wild introgression in Asia, although not necessarily from Argali or Urial sheep (supplementary fig. S11, Supplementary Material online) and/or contribution from an undocumented independently domesticated group. These admixed sheep lineages must then have spread and admixed with local breeds due to some desired traits, akin to rapid introgression of Zebu across Asian cattle (Verdugo et al. 2019). Our data suggest that this may have happened over the last two millennia as the Baltic and Kalmykia genomes do not appear to carry this unique ancestry. It is noteworthy that analyses of modern-day genotypes have also suggested recent common ancestry among sheep breeds, going back only 800 generations (∼3,200 years) (Ezard et al. 2009; Kijas et al. 2012). We speculate that highly beneficial traits, such as a superior wool variety, high-fat content, or disease resistance, might have facilitated such rapid admixture. It would be attractive to test this using high-resolution time transect data (Cheng et al. 2023).

## Conclusion

Our findings rectify and resolve several issues related to the domestication and post-domestication history of sheep. First, the data from Anatolian Epipaleolithic sheep as well as modern-day Anatolian and Cyprian Mouflons mark a likely Anatolian source of domestic sheep, which could include both Central and Southeast Anatolian/upper Mesopotamian wild sheep populations in line with zooarchaeological observations (Peters et al. 2005; Hongo et al. 2009; Arbuckle and Atıcı 2013; Baird 2014; Stiner et al. 2014, 2022; Abell et al. 2019). Meanwhile, the limited structure among domestic sheep across Neolithic-SW Asia leaves open the question of whether sheep originated from a single domestication event or had multiple domestication centers as in goats and cattle (Daly et al. 2018; Verdugo et al. 2019). That said, given our results from the analysis of Anatolian Epipaleolithic sheep, we can safely assume that Central Anatolian sheep were part of the first domesticated gene pool.

Second, we find widespread evidence of sheep lineages being admixed with each other as well as with wild sheep through the Holocene, including the apparent replacement of early 7th millennium BCE Central Anatolian sheep, Asian domestic admixture into 2nd millennium BCE Baltic sheep, and a near-ubiquitous ancestry that appears to have spread globally after the Bronze Age, that we speculate may be related to the secondary product dispersal (Sherratt 1981; Vigne and Helmer 2007; Greenfield 2010). This picture raises the question of whether sheep may have been transported and/or admixed more intensely than most other domestic species.

Finally, we note instances where the demographic history of sheep appears to mirror that of humans, resembling parallel patterns of human and dog mobility reported recently (Bergström et al. 2020). We had earlier noted such parallel changes for the Mediterranean, where Neolithic Anatolian sheep and humans appear genetically closer to those of present-day South Europe than those of present-day Anatolia (Yurtman et al. 2021). Our findings now expand these patterns, including the observed loss of diversity in humans and sheep associated with the Mediterranean expansion via the coastal route, or the eastern admixtures into North European sheep and humans during the Bronze Ages. However, the more recent admixture event that we infer, which appears to have shaped the global sheep gene pool within the last two millennia, does not appear to have parallels in human demographic history, suggesting that sometimes the sheep moved while the shepherds stayed.

## Materials and Methods

### Descriptions of Sample Collection Sites and Samples

In the present study, we screened *n* = 24 archeological bone samples from Anatolia (*n* = 15), Iran (*n* = 2), Russia (*n* = 1), Iberia (*n* = 2), France (*n* = 3), and Baltic (*n* = 1). Among these, 17 were produced in this study, three were produced in a parallel study (Morell Miranda 2023; Larsson et al. 2024) while four Anatolian Neolithic samples from our previous publication (Yurtman et al. 2021) were further deep-sequenced (Fig. 1) (see Supplementary material for detailed descriptions of archeological sites and samples). Anatolian ancient samples were collected from seven archeological sites, one of which is an Epipaleolithic rock shelter named Pınarbaşı (*n* = 1). The remaining six sites are Neolithic settlements from West and Central Anatolia as well as the Lakes District. West Anatolian sites are Ulucak Höyük (*n* = 3) and Barcın Höyük (*n* = 1) whereas Central Anatolian Neolithic samples are from Tepecik-Çiftlik Höyük (*n* = 6). Iranian Neolithic samples were obtained from Tepe Khaleseh, a late Neolithic site in northwest Iran, whereas Russian samples are from the Iron Age site Zahanata in Kalmykia. Iberian Neolithic samples were from El Portalón de Cueva Mayor a cave in Northern Iberia. The early French Neolithic samples originate from the archeological sites in Menneville-Derrière-Le-Village and Bucy-le-Long “le Fond du Petit Marais” in Picardie, Northern France. The Baltic sample was excavated from the cave Stora Förvar on Stora Karlsö, an island near Gotland, a location with potential connections to both sides of the Baltic Sea.

Anatolian Mouflon blood samples were collected from the remaining herd with less than 500 individuals in the Konya region of Central Anatolia, with the approval of the Selçuk University Veterinary Faculty Ethics Committee (permit number: 2009/041) and were collected by the General Directorate of Nature Conservation and National Parks, Turkish Republic Ministry of Forestry and Hydraulic Works. The samples were studied with the permission of the institution (permit number: 797 dated 2009/12/22) and with the approval of the Local Committee on the Ethics of Animal Experiments of the Middle East Technical University (permit number: 2009/18). Cyprian Mouflon tissues were collected from the individuals found dead in the Pafos Forest Reserve on the northwest slopes of the Troodos Mountains, under the permit of the Ministry of the Interior for scientific research.

### Radiocarbon Dating

We AMS C14 dated one bone (zah001 from Kalmykia, Russia) at the TÜBİTAK MAM (Gebze, Ankara). Iberian Neolithic (APOR008) and Baltic Neolithic (ASTF001) samples were radiocarbon-dated by the Tandem Laboratory at Uppsala University. APOR009 was contextually dated by a seed found at the same quadrant and layer. Bones and teeth were mechanically cleaned by scraping the surface and then ground in a mortar. The samples were incubated with 0.25 M HCl at ambient temperature for 48 h. A total of 0.01 M HCl was added to the insoluble fraction and incubated at 50 °C for 16 h. The soluble fraction was added to a 30 kDa ultrafilter and centrifuged, and the retentate was lyophilized. Before determination, the fraction to be dated was combusted to CO_2_ using a Fe-catalyst. Acquired dates were calibrated using OxCal 4.4 (Ramsey 2009) using IntCal20 (Reimer et al. 2020) as the calibration curve.

### Modern DNA Extraction

DNA from Anatolian Mouflon whole blood samples (*N* = 5) was extracted using a standard phenol: chloroform extraction protocol (Sambrook et al. 1989). For the Cyprian Mouflons (*N* = 2), DNA was extracted from tissues using the NucleoSpin tissue kit, following the standard protocol. For both sets of samples, DNA isolates were fragmented through sonication with Qsonica Q800R at 100% amplitude for 15 s on/15 s off at 4 °C for a total of 12 min.

### Ancient Sample Preparation and DNA Extraction

#### METU Lab

All experimental procedures were carried out in dedicated ancient DNA laboratories. All necessary measures were taken to minimize contamination. Laboratory equipment was decontaminated with DNAaway while benches and other surfaces were cleaned with 2% NaOCl. We included negative controls during the DNA isolation, library preparation, and PCR amplification steps. First, the outer surfaces of the bone or teeth samples were cleaned off the soil or other exogenous contaminants with a sandblasting cutting disk attached to the Dremel tool. Next, we cut out a small piece of bone from each sample and exposed each side of the bone to UV for 15 min. UV-exposed bones were ground to obtain bone powder. DNA extractions were performed following Dabney et al. (2013).

#### Institut Jacques Monod-Paris Lab

DNA extraction, purification, and DNA library preparation were performed as described before (Bennett et al. 2022, 2023). Briefly, the temporal bones were cleaned through wiping with water, concentrated bleach, and water again. The densest parts of the petrous bones were cut using a flame-sterilized diamond disc of a Dremel saw. A small part of the bone was ground to fine powder in liquid nitrogen in a 6775 Freezer/MillSpex SamplePrep. The bone powder was washed with phosphate buffer according to Korlević et al. (2015). DNA extraction was performed by incubating the bone powder at 37 °C for 72 h in twice 1 ml extraction buffer B (0.5 M ethylenediaminetetraacetic acid, 0.05% Tween-20, 250 μg/ml Proteinase K, 0.14 M β-mercaptoethanol) that were pooled prior to purification. Samples were purified using silica membrane spin-columns (QIAquick Gel Extraction kit) with a vacuum manifold (Qiagen) and 25 ml extenders (Qiagen) as described (Gorgé et al. 2023) using the 5 M guanidine HCl, 40% isopropanol (5M40) buffer as described in Dabney et al. (2013). The elution was performed twice in 25 μl 10 mM Tris-HCl pH 8.0, 0.05% Tween-20 made from gamma-irradiated water (8 kGy).

#### Uppsala Lab

DNA extraction and library preparation were done at the dedicated aDNA facilities of the SciLifeLab Ancient DNA Unit, in Uppsala, Sweden. Before extraction, samples were irradiated with UV light (6 J/cm^2^ at 254 nm) for 20 min, and then their outer surface was removed using a Dremel drill. Samples were then wiped with sterile cotton swabs with 0.5% sodium hypochlorite solution and UV-irradiated Mili-Q water and exposed to UV-irradiation again on each surface. Then, a Dremel tool cut subsamples of 50 to 100 mg from each sample. DNA was extracted using a modified silica-based method (Meyer and Kircher 2010; Dabney et al. 2013). Instead of sodium dodecyl sulfate, 1 M urea was used in the extraction buffer. For every ten samples, one extraction blank was added as a negative control. Subsamples were pretreated with 1 ml of 0.5 M ethylenediaminetetraacetic acid (Invitrogen) and incubated at 37 °C for 30 min. The ethylenediaminetetraacetic acid solution was removed, and the subsample was digested with 1 ml extraction buffer (0.44 M ethylenediaminetetraacetic acid/1 M urea) containing 0.25 mg/ml protein kinase K (Sigma-Aldrich). They were incubated with rotation for ∼23 h at 37 °C and then at 55 °C for 6.5 h. The supernatant was collected and stored at −20 °C. One ml of fresh extraction buffer with protein kinase was added to the sample and incubated further at 55 °C for 19 h. Supernatants were combined and concentrated using an Amicon Ultra-4 filter unit (Millipore). DNA was purified using MinElute PCR purification kits (Qiagen) and eluted in a total volume of 110 μl EB buffer. Qubit dsDNA HS assay (Invitrogen) was used to determine the concentration of the DNA extract.

### Whole Genome Library Preparation and Prescreening

#### METU Lab

Double-stranded, blunt-end, double-indexed Illumina compatible whole genome libraries (*N* = 238) were prepared following (Kircher 2012) protocol and sequenced on Illumina Novaseq 6000 S1 or S4 flowcells (median of c. 26 million reads per sample). Out of 231 ancient libraries, 18 were found to contain >0.4% endogenous DNA, which was further sequenced on Illumina Novaseq 6000 S1 or S4.

#### Institut Jacques Monod-Paris Lab

Libraries were constructed using the NEBNext Ultra II DNA Library Prep Kit for Illumina (NEB, Ipswich, MA, USA) after a pretreatment with USER enzyme mix (NEB, Ipswich, MA, USA), as described before (Bennett et al. 2022, 2023). Dual-barcoded libraries were then purified and size-selected using NucleoMag beads (Macherey-Nagel) for two rounds of purification following the supplied protocol at a ratio of 1.3× beads per reaction volume and eluted in 30 μl EBT. All libraries were quantified with a Qubit 2.0 Fluorometer (Thermo Fisher Scientific), Bioanalyzer 2100 (Agilent, Santa Clara, CA, USA), and by qPCR. Screening by shotgun sequencing was performed on an Illumina MiSeq system using a v3 reagent kit for 2 × 75 cycles.

#### Uppsala Lab

Double-stranded blunt-end libraries were prepared from 20 μl of DNA extraction according to protocol, with some modifications (Meyer and Kircher 2010) (MineElute PCR purification kits were used to clean enzymatic reactions instead of SPRI beads). For every ten samples, an extraction and library blank were added as a negative control. Libraries were quantified by real-time qPCR in a 20 μl reaction using Maxima SYBR green master mix (Thermo Fisher Scientific), 200 nM IS7 primer, and 200 nM IS8 primer, to determine the number of indexing cycles. Dual-indexing PCR amplification was performed in duplicates in a 50 μl reaction using 6 μl DNA library, 5 U Ampli-Taq Gold DNA polymerase (Thermo Fisher Scientific), 1× GeneAmpl GIs 244 cIs old Buffer (Thermo Fisher Scientific), 2.5 mM MgCl_2_, 250 μM of each dNTP, 200 nM P7 indexing primer, and 200 nM P5 indexing primer (Meyer and Kircher 2010; Kircher 2012). The PCR reaction was performed at 94 °C for 10 min, 13 to 20 cycles of (94 °C for 30 s, 60 °C for 30 s, 72 °C for 45 s), and 72 °C for 10 min. Duplicates were pooled and purified with AMPure XP beads (Beckman Coulter). The quality of the libraries was analyzed by the 2200 Tapestation System (Agilent), and the Qubit dsDNA HS assay (Invitrogen) was used for the quantification of the sequencing libraries.

### Data Preprocessing and SNP Dataset Preparation

#### Ancient DNA Data Preprocessing

For each library, residual adapter sequences in the raw FASTQ files were eliminated using “Adapter Removal” software (version 2.3.1) (Schubert et al. 2016) with parameters “--qualitybase 33 --gzip --trimns” and a minimum 11 bp overlap between pairs “--collapse --minalignmentlength 11”. Then, paired-end sequenced fastq files were merged. The merged reads were aligned to the sheep reference genome Oar v.4.0 (https://www.ncbi.nlm.nih.gov/assembly/GCF_000298735.2) using “BWA aln/samse” (version 0.7.15) (Li and Durbin 2009) with parameters “-n 0.01, -o 2” and the seed disabled with “-l 16500.” Multiple libraries from the same individual were merged with “samtools merge” (version 1.9) (Danecek et al. 2021), and PCR duplicates with identical start and end coordinates were removed using “FilterUniqueSAMCons.py” (Kircher 2012). Reads with >10% mismatches to the sheep reference genome, and <35 base pairs were also excluded. Finally, we applied a mapping quality > 30. The average genome coverage was calculated using “genomeCoverageBed” within “bedtools2” (Quinlan and Hall 2010). MTaxi (Atağ et al. 2022) was used to confirm the taxa of low coverage samples (supplementary table S1, Supplementary Material online).

#### Modern DNA Data Preprocessing

For each library, residual adapter sequences in the raw FASTQ files were removed using the same methods and parameters used in the ancient data. However, this time, the --collapse parameter was not used and paired-end sequenced fastq files were left as is, without being merged. The paired-end reads were mapped onto the sheep reference genome Oar v.4.0 using “BWA-mem” (version 0.7.15) (Li 2013) with the default parameters. To remove duplicate reads, the program “Picard MarkDuplicates” (http://broadinstitute.github.io/picard/) was used. Finally, we applied a mapping quality > 20 filters to aligned bam files.

#### SNP Dataset Preparation

We used nine published modern-day wild genomes (*O. ammon*, *O. a. polii*, *O. canadensis*, *O. dalli*, *O. nivicola*) with coverages ranging from 7.4 to 16.5× to identify a total of 15,929,043 SNPs using the “Genome Analysis Toolkit (GATK)” v.4.0.11.0 (McKenna et al. 2010). The reason for using these lineages for de novo SNP determination was that they are supposed to be true outgroups to the domestic sheep. By determining SNPs in outgroup lineages, our motivation was to avoid ascertainment bias (Wang and Nielsen 2012) toward certain modern-day breeds, which can skew diversity and genetic similarity estimates. After employing HaplotypeCaller, CombineGVCFs, and GenotypeGVCFs commands for variant combination and genotyping by using the “--min-base-quality-score 30” parameter, we utilized “bcftools” (Li 2011) for the removal of multiallelic positions. Further, we applied “--maf 0.05 --hwe 0.001” filtering using “vcftools” (Danecek et al. 2011) to remove SNPs with minor allele frequency < 0.05 and deviating from Hardy–Weinberg equilibrium at *P* < 0.001. After this filtering process, a total of 10,343,589 SNP positions were obtained. These de novo SNPs positions were used for diploid genotyping 185 genomes of published modern-day domestic sheep breeds **(**supplementary table S2, Supplementary Material online) by using GATK HaplotypeCaller tools using settings: “--genotyping-mode GENOTYPE_GIVEN_ALLELES” “--output-mode EMIT_ALL_SITES”. Next, we used a hard filtering process by applying the following criteria: “QD < 2.0, FS > 60.0, MQ < 40.0, SOR > 3.0, QUAL < 30.0, MQRankSum < −12.5, ReadPosRankSum > −8.0” using “bcftoos” (Li 2011), following the workflow in Li et al. (2020). We then converted the vcf dataset format to the plink data format by using the “plink2” tool (Chang et al. 2015). SNP positions with less than 5 bp between each other were removed from the dataset, and we further applied the filters “--hwe 0.001”, “--geno 0.05”, “--mind 0.1”, thus filtering out SNPs out of Hardy–Weinberg equilibrium (possible paralogs) and SNPs with low genotyping rate. In the end, a total of 4,617,899 autosomal SNP positions were obtained.

#### Postmortem Damage (PMD) Removal

Postmortem deamination patterns were determined from BAM files using PMDtools (Skoglund et al. 2014) with the “--deamination” parameter. To eliminate these deamination patterns, we applied trimming 10 base pairs from both ends (except for the French Neolithic samples which were trimmed only 2 bases since they were USER-treated) using the “bam” command within the “bamUtil” software (Jun et al. 2015).

### Genotyping and Data Analysis

#### Genotyping

We used two different approaches for ancient and modern genomes for genotyping. To prevent genotype calling biases arising from variations in sequencing coverage across samples, we pseudohaploidized the ancient data. This involved the random selection of one allele for each SNP position, accomplished through the genotype caller “pileupCaller” (version 1.5.3.1) (https://github.com/stschiff/sequenceTools) applied to the output pileup file of “samtools mpileup” (with base quality > 30 and MAPQ > 30) (Danecek et al. 2021). For modern-day genomic data, we performed diploid genotype calling using “GATK HaplotypeCaller” (version 4.0.11.0) (McKenna et al. 2010) with the “--genotyping-mode GENOTYPE_GIVEN_ALLELES”, “--output-mode EMIT_ALL_SITES” parameters and the “--alleles” parameters. This alleles list was obtained from de novo SNP positions ascertained in wild sheep.

For the genotyping process by using 50 K BeadChip (https://figshare.com/articles/dataset/Mapping_of_ISGC_SNP_chip_probes/8424935/2), we initially performed diploid genotyping on 185 genomes from published modern domestic sheep breeds (supplementary table S2, Supplementary Material online). This involved using the “bcftools mpileup (v 1.18)” command with parameters “-I -E -T -q 30 -Q 30” followed by “bcftools call -Aim -C alleles” (Li 2011). Here, we utilized Illumina OvineSNP50 Beadchip SNP positions based on the Oar v.4.0 reference genome coordinates. Additionally, for ancient individuals, we created pseudo-haploid datasets by randomly selecting one allele per targeted SNP position using the “pileupCaller” (version 1.5.3.1) genotype caller. Finally, we merged this ancient sample data with genotype information from the 185 domestic sheep breeds data.

#### Mitogenome Assembly and Haplogroup Inference

The Mapping Iterative Assembler (MIA) (version 5a7fb5a) (https://github.com/udo-stenzel/mapping-iterative-assembler) was used to assemble consensus sequences for the mitogenomes of ancient samples. To reduce references bias, we used as reference an Iranian Mouflon mitochondrial mitogenome (NCBI Reference Sequence NC-026063), as well as a custom substitution matrix that takes into account postmortem damage. Consensus sequences were assembled using only sites with a minimum coverage of 10×, a minimum quality of 40, and two-thirds base agreement on each position. Any site that did not pass these filters was set to “N”. The haplogroup of all sequences with enough information to be assembled was then inferred using MitoToolsPy (version 1.0) (Fan and Yao 2011) using the sheep reference and the whole mitochondria.

#### Molecular Sex Determination

We determined the genetic sex of the studied genomes utilizing the *Rx* metric (Mittnik et al. 2016) thresholds optimized for sheep, using SexDetermineOar (https://github.com/mskilic/SexDetermineOar).

#### Diversity Estimates

We calculated within-population diversity levels using 1 − outgroup *f*_3_ values with the ascertained SNP panel, including only transversions (Fig. 6), or with both transitions and transversions (supplementary fig. S6, Supplementary Material online). *f*_3_ calculations were performed with goat as outgroup using the python program POPSTATS (Skoglund et al. 2015) with “--f3vanilla” (for the simple *f*_3_ statistic *f*_3_ = (p3 − p1)(p3 − p2)) and “--not23” options (to use provided chromosomes in the input file, the latter option supports non-human organisms). Statistical significance of diversity differences between groups was tested using Kruskal–Wallis test and the Wilcoxon rank sum test.

#### Principal Component Analysis

PCA was conducted using EIGENSOFT (v.7.2.0) (Patterson et al. 2006) “smartpca” command with the “lsqproject: YES” parameter. Components of individuals from published modern populations were first calculated (supplementary table S3, Supplementary Material online), and ancient individuals were projected onto the first two components. Visualization of the PCA was done by the R (v.4.3.1) (R Core Team 2023) library ggplot2 (Wickham 2016).

#### *D*-statistics

*D*-statistics were calculated using angsd (v.0.938) (Korneliussen et al. 2014) “ABBABABA (multipop)” command. *D*-statistic results were visualized using R (v.4.3.1) (R Core Team 2023) library ggplot2 (Wickham 2016).

#### *D*-PCA

A PCA based on *D*-statistics (Bergström et al. 2020) was performed by first calculating all possible combinations of *D*-statistics of the form *D(A, B; C, D)*, using only transversion SNPs, 1,000,000 blocksize, -minQ 30 -minMapQ 30 options, and running the analysis for only autosomes. PCA components were calculated using the *prcomp* function and visualized by plotting the first two eigenvectors of PCA by the ggplot2 package.

#### *f*4-Ratio Analysis

To understand the admixture proportions in the Baltic Neolithic sample, *f*4-ratio (as *alpha*=*f4*(A,O;X,C)/*f4*(A,O;B,C)) was calculated using ADMIXTOOLS software qpF4ratio module (Patterson et al. 2012). We used the model *f4(Iran_N, Goat; Baltic_N, Iberia_N/France_N) / f4(Iran_N, Goat; Kyrgyzstan_N, Iberia_N/France_N)*.

## Supplementary Material

msae158_Supplementary_Data

## Data Availability

All FASTQ files were submitted to the European Nucleotide Archive (ENA) with reference numbers PRJEB69690 and PRJEB81145.

## References

[msae158-B1] Abell JT, Quade J, Duru G, Mentzer SM, Stiner MC, Uzdurum M, Özbaşaran M. Urine salts elucidate early Neolithic animal management at Aşıklı Höyük, Turkey. Sci Adv. 2019:5(4):eaaw0038. 10.1126/sciadv.aaw0038. 31001590 PMC6469938

[msae158-B2] Allentoft ME, Sikora M, Sjögren K-G, Rasmussen S, Rasmussen M, Stenderup J, Damgaard PB, Schroeder H, Ahlström T, Vinner L, et al Population genomics of Bronze Age Eurasia. Nature. 2015:522(7555):167–172. 10.1038/nature14507. 26062507

[msae158-B3] Altınışık NE, Kazancı DD, Aydoğan A, Gemici HC, Erdal ÖD, Sarıaltun S, Vural KB, Koptekin D, Gürün K, Sağlıcan E, et al A genomic snapshot of demographic and cultural dynamism in Upper Mesopotamia during the Neolithic Transition. Sci Adv. 2022:8(44):eabo3609. 10.1126/sciadv.abo3609. 36332018 PMC9635823

[msae158-B4] Arbogast RM . Premiers élevages néolithiques du Nord-Est de la France. Liège: Université de Liège; 1994.

[msae158-B5] Arbuckle BS, Atici L. Initial diversity in sheep and goat management in Neolithic south-western Asia. Levant. 2013:45(2):219–235. 10.1179/0075891413Z.00000000026.

[msae158-B6] Arbuckle BS, Kansa SW, Kansa E, Orton D, Çakırlar C, Gourichon L, Atici L, Galik A, Marciniak A, Mulville J, et al Data sharing reveals complexity in the westward spread of domestic animals across Neolithic Turkey. PLoS One. 2014:9(6):e99845. 10.1371/journal.pone.0099845. 24927173 PMC4057358

[msae158-B7] Atağ G, Kaptan D, Yüncü E, Başak Vural K, Mereu P, Pirastru M, Barbato M, Leoni GG, Güler MN, Er T, et al Population genomic history of the endangered Anatolian and Cyprian Mouflons in relation to worldwide wild, feral, and domestic sheep lineages. Genome Biol Evol. 2024:16(5):evae090. 10.1093/gbe/evae090. 38670119 PMC11109821

[msae158-B8] Atağ G, Vural K, Kaptan D, Özkan M, Koptekin D, Sağlıcan E, Doğramacı S, Köz M, Yılmaz A, Söylev A, et al MTaxi: a comparative tool for taxon identification of ultra low coverage ancient genomes. Open Res Europe. 2022:2:100. 10.12688/openreseurope.14936.1. PMC10565424 37829208

[msae158-B9] Auxiette G, Hachem L. Farm, hunt, feast, celebrate. Animals and society in Neolithic Bronze and Iron Age northern France. Leiden: Sidestone Press; 2021.

[msae158-B10] Baird D . Origins of caprine herding. Proc Natl Acad Sci USA. 2014:111(24):8702–8703. 10.1073/pnas.1406870111. 24927556 PMC4066497

[msae158-B11] Baird D, Fairbairn A, Jenkins E, Martin L, Middleton C, Pearson J, Asouti E, Edwards Y, Kabukcu C, Mustafaoğlu G, et al Agricultural origins on the Anatolian plateau. Proc Natl Acad Sci USA. 2018:115(14):E3077–E3086. 10.1073/pnas.1800163115. 29555740 PMC5889673

[msae158-B12] Barbato M, Hailer F, Orozco-terWengel P, Kijas J, Mereu P, Cabras P, Mazza R, Pirastru M, Bruford MW. Genomic signatures of adaptive introgression from European mouflon into domestic sheep. Sci Rep. 2017:7(1):7623. 10.1038/s41598-017-07382-7. 28790322 PMC5548776

[msae158-B13] Bennett EA, Parasayan O, Prat S, Péan S, Crépin L, Yanevich A, Grange T, Geigl E-M. Genome sequences of 36,000- to 37,000-year-old modern humans at Buran-Kaya III in Crimea. Nat Ecol Evol. 2023:7(12):2160–2172. 10.1038/s41559-023-02211-9. 37872416

[msae158-B14] Bennett EA, Weber J, Bendhafer W, Champlot S, Peters J, Schwartz GM, Grange T, Geigl E-M. The genetic identity of the earliest human-made hybrid animals, the kungas of Syro-Mesopotamia. Sci Adv. 2022:8(2):eabm0218. 10.1126/sciadv.abm0218. 35030024 PMC8759742

[msae158-B15] Bergström A, Frantz L, Schmidt R, Ersmark E, Lebrasseur O, Girdland-Flink L, Lin AT, Storå J, Sjögren K-G, Anthony D, et al Origins and genetic legacy of prehistoric dogs. Science. 2020:370(6516):557–564. 10.1126/science.aba9572. 33122379 PMC7116352

[msae158-B16] Blyth E . XXVI.—an amended list of the species of the genus *Ovis*. J Nat Hist. 1841:7(43):195–201. 10.1080/03745484109442689.

[msae158-B17] Brigand R, Dubouloz J, Weller O. Colonization dynamics of LBK farmers in Europe under geostatistics test. Doc Praehist. 2022:49:2–45. 10.4312/dp.49.12.

[msae158-B18] Bruford MW, Townsend SJ. Mitochondrial DNA diversity in modern sheep. In: Zeder MA, Bradley D, Emshwiller E, Smith BD, editors. Documenting domestication: new genetic and archaeological paradigms. Berkeley: University of California Press; 2006. p. 306–316.

[msae158-B19] Cai D, Zhang N, Shao X, Sun W, Zhu S, Yang DY. New ancient DNA data on the origins and spread of sheep and cattle in northern China around 4000 BP. Asian Archaeol. 2018:2(1):51–57. 10.1007/s41826-018-0018-z.

[msae158-B20] Chang CC, Chow CC, Tellier LC, Vattikuti S, Purcell SM, Lee JJ. Second-generation PLINK: rising to the challenge of larger and richer datasets. Gigascience. 2015:4(1):s13742-015. 10.1186/s13742-015-0047-8. PMC4342193 25722852

[msae158-B21] Chataigner C, Badalyan R, Arimura M. The Neolithic of the Caucasus. Oxford Handbooks Online; 2014.

[msae158-B22] Chen Z-H, Xu Y-X, Xie X-L, Wang D-F, Aguilar-Gómez D, Liu G-J, Li X, Esmailizadeh A, Rezaei V, Kantanen J, et al Whole-genome sequence analysis unveils different origins of European and Asiatic mouflon and domestication-related genes in sheep. Commun Biol. 2021:4(1):1307. 10.1038/s42003-021-02817-4. 34795381 PMC8602413

[msae158-B23] Cheng H, Zhang Z, Wen J, Lenstra JA, Heller R, Cai Y, Guo Y, Li M, Li R, Li W, et al Long divergent haplotypes introgressed from wild sheep are associated with distinct morphological and adaptive characteristics in domestic sheep. PLoS Genet. 2023:19(2):e1010615. 10.1371/journal.pgen.1010615. 36821549 PMC9949681

[msae158-B24] Chessa B, Pereira F, Arnaud F, Amorim A, Goyache F, Mainland I, Kao RR, Pemberton JM, Beraldi D, Stear MJ, et al Revealing the history of sheep domestication using retrovirus integrations. Science. 2009:324(5926):532–536. 10.1126/science.1170587. 19390051 PMC3145132

[msae158-B25] Ciani E, Mastrangelo S, Da Silva A, Marroni F, Ferenčaković M, Ajmone-Marsan P, Baird H, Barbato M, Colli L, Delvento C, et al On the origin of European sheep as revealed by the diversity of the Balkan breeds and by optimizing population-genetic analysis tools. Genet Sel Evol. 2020:52(1):25. 10.1186/s12711-020-00545-7. 32408891 PMC7227234

[msae158-B26] Clutton-Brock J . Domesticated animals from early times. Austin (TX): University of Texas Press; 1981.

[msae158-B27] Colledge S, Conolly J, Shennan S. The evolution of Neolithic farming from SW Asian origins to NW European limits. Eur J Archaeol. 2005:8(2):137–156. 10.1177/1461957105066937.

[msae158-B28] Dabney J, Meyer M, Pääbo S. Ancient DNA damage. Cold Spring Harb Perspect Biol. 2013:5(7):a012567. 10.1101/cshperspect.a012567. 23729639 PMC3685887

[msae158-B29] Daly KG, Maisano Delser P, Mullin VE, Scheu A, Mattiangeli V, Teasdale MD, Hare AJ, Burger J, Verdugo MP, Collins MJ, et al Ancient goat genomes reveal mosaic domestication in the Fertile Crescent. Science. 2018:361(6397):85–88. 10.1126/science.aas9411. 29976826

[msae158-B30] Danecek P, Auton A, Abecasis G, Albers CA, Banks E, DePristo MA, Handsaker RE, Lunter G, Marth GT, Sherry ST, et al The variant call format and VCFtools. Bioinformatics. 2011:27(15):2156–2158. 10.1093/bioinformatics/btr330. 21653522 PMC3137218

[msae158-B31] Danecek P, Bonfield JK, Liddle J, Marshall J, Ohan V, Pollard MO, Whitwham A, Keane T, McCarthy SA, Davies RM, et al Twelve years of SAMtools and BCFtools. Gigascience. 2021:10(2):giab008. 10.1093/gigascience/giab008. 33590861 PMC7931819

[msae158-B32] Demirci S, Baştanlar EK, Dağtaş ND, Pişkin E, Engin A, Özer F, Yüncü E, Doğan ŞA, Togan İ. Mitochondrial DNA diversity of modern, ancient and wild sheep (*Ovis gmelinii anatolica*) from Turkey: new insights on the evolutionary history of sheep. PLoS One. 2013:8(12):e81952. 10.1371/journal.pone.0081952. 24349158 PMC3859546

[msae158-B33] Deng J, Xie X-L, Wang D-F, Zhao C, Lv F-H, Li X, Yang J, Yu J-L, Shen M, Gao L, et al Paternal origins and migratory episodes of domestic sheep. Curr Biol. 2020:30(20):4085–4095.e6. 10.1016/j.cub.2020.07.077. 32822607

[msae158-B34] Ezard TH, Côté SD, Pelletier F. Eco-evolutionary dynamics: disentangling phenotypic, environmental and population fluctuations. Philos Trans R Soc B Biol Sci. 2009:364(1523):1491–1498. 10.1098/rstb.2009.0006. PMC2690502 19414464

[msae158-B35] Fan L, Yao Y-G. MitoTool: a web server for the analysis and retrieval of human mitochondrial DNA sequence variations. Mitochondrion. 2011:11(2):351–356. 10.1016/j.mito.2010.09.013. 20933105

[msae158-B36] Garcia-Suarez A, Portillo M, Matthews W. Early animal management strategies during the Neolithic of the Konya Plain, Central Anatolia: integrating micromorphological and microfossil evidence. Environ Archaeol. 2020:25(2):208–226. 10.1080/14614103.2018.1497831.

[msae158-B37] Gorgé O, Bennett EA, Massilani D, Daligault J, Geigl E-M, Grange T. Analysis of ancient microbial DNA. Methods Mol Biol. 2023:(2605):103–131. 10.1007/978-1-0716-2871-3_6. 36520391

[msae158-B38] Greenfield HJ . The secondary products revolution: the past, the present and the future. World Archaeol. 2010:42(1):29–54. 10.1080/00438240903429722.

[msae158-B39] Haak W, Lazaridis I, Patterson N, Rohland N, Mallick S, Llamas B, Brandt G, Nordenfelt S, Harney E, Stewardson K, et al Massive migration from the steppe was a source for Indo-European languages in Europe. Nature. 2015:522(7555):207–211. 10.1038/nature14317. 25731166 PMC5048219

[msae158-B40] Hachem L . Le site néolithique de Cuiry-lès-Chaudardes – I. De l'analyse de la faune à la structuration sociale. Verlag Marie Leidorf, Rahden/Westf: Internationale Archäologie; 2011.

[msae158-B41] Hachem L . Animals in LBK society: identity and gender markers. J Archaeol Sci: Rep. 2018:20:910–921. 10.1016/j.jasrep.2017.09.020.

[msae158-B42] Hadjisterkotis E . The Cyprus mouflon Ovis gmelini ophion: management, conservation and evolution. Montréal (Québec), Canada: McGill University; 1992.

[msae158-B43] Harris DR . Origins and spread of agriculture and pastoralism in Eurasia. Smithsonian Institution Press; 1996. 10.9783/9781934536513.

[msae158-B44] Harris DR, Gosden C. The beginnings of agriculture in western Central Asia. In: The origins and spread of agriculture and pastoralism in Eurasia. University of Pennsylvania Press; 2010. 10.9783/9781934536513.225.

[msae158-B45] Her C, Rezaei H, Hughes S, Naderi S, Duffraisse M, Mashkour M, Naghash H, Bălășescu A, Luikart G, Jordan S, et al Broad maternal geographic origin of domestic sheep in Anatolia and the Zagros. Anim Genet. 2022:53(3):452–459. 10.1111/age.13191. 35288946

[msae158-B46] Hiendleder S, Mainz K, Plante Y, Lewalski H. Analysis of mitochondrial DNA indicates that domestic sheep are derived from two different ancestral maternal sources: no evidence for contributions from urial and argali sheep. J Hered. 1998:89(2):113–120. 10.1093/jhered/89.2.113. 9542158

[msae158-B47] Hongo H, Pearson J, Öksüz B, Ilgezdi G. The process of ungulate domestication at Çayönü, Southeastern Turkey: a multidisciplinary approach focusing on *Bos* sp. and *Cervus elaphus*. Anthropozoologica. 2009:44(1):63–78. 10.5252/az2009n1a3.

[msae158-B48] Jun G, Wing MK, Abecasis GR, Kang HM. An efficient and scalable analysis framework for variant extraction and refinement from population-scale DNA sequence data. Genome Res. 2015:25(6):918–925. 10.1101/gr.176552.114. 25883319 PMC4448687

[msae158-B49] Kijas JW, Lenstra JA, Hayes B, Boitard S, Porto Neto LR, San Cristobal M, Servin B, McCulloch R, Whan V, Gietzen K, et al Genome-wide analysis of the world's sheep breeds reveals high levels of historic mixture and strong recent selection. PLoS Biol. 2012:10(2):e1001258. 10.1371/journal.pbio.1001258. 22346734 PMC3274507

[msae158-B50] Kijas JW, Townley D, Dalrymple BP, Heaton MP, Maddox JF, McGrath A, Wilson P, Ingersoll RG, McCulloch R, McWilliam S, et al A genome wide survey of SNP variation reveals the genetic structure of sheep breeds. PLoS One. 2009:4(3):e4668. 10.1371/journal.pone.0004668. 19270757 PMC2652362

[msae158-B51] Kircher M . Analysis of high-throughput ancient DNA sequencing data. Methods Mol Biol. 2012:840:197–228. 10.1007/978-1-61779-516-9_23. 22237537

[msae158-B52] Korlević P, Gerber T, Gansauge MT, Hajdinjak M, Nagel S, Aximu-Petri A, Meyer M. Reducing microbial and human contamination in DNA extractions from ancient bones and teeth. Biotechniques. 2015:59(2):87–93. 26260087 10.2144/000114320

[msae158-B53] Korneliussen TS, Albrechtsen A, Nielsen R. ANGSD: analysis of next generation sequencing data. BMC Bioinformatics. 2014:15(1):356. 10.1186/s12859-014-0356-4. 25420514 PMC4248462

[msae158-B54] Larson G, Piperno DR, Allaby RG, Purugganan MD, Andersson L, Arroyo-Kalin M, Barton L, Climer Vigueira C, Denham T, Dobney K, et al Current perspectives and the future of domestication studies. Proc Natl Acad Sci USA. 2014:111(17):6139–6146. 10.1073/pnas.1323964111. 24757054 PMC4035915

[msae158-B55] Larsson MN, Morell Miranda P, Pan L, Başak Vural K, Kaptan D, Rodrigues Soares AE, Kivikero H, Kantanen J, Somel M, Özer F. Ancient sheep genomes reveal four Millennia of North European short-tailed sheep in the Baltic Sea region. Genome Biol Evol. 2024:16(6). 10.1093/gbe/evae114. PMC11162877 38795367

[msae158-B56] Lazaridis I, Alpaslan-Roodenberg S, Acar A, Açıkkol A, Agelarakis A, Aghikyan L, Akyüz U, Andreeva D, Andrijašević G, Antonović D, et al Ancient DNA from Mesopotamia suggests distinct pre-pottery and pottery Neolithic migrations into Anatolia. Science. 2022:377(6609):982–987. 10.1126/science.abq0762. 36007054 PMC9983685

[msae158-B57] Li H . A statistical framework for SNP calling, mutation discovery, association mapping and population genetical parameter estimation from sequencing data. Bioinformatics. 2011:27(21):2987–2993. 10.1093/bioinformatics/btr509. 21903627 PMC3198575

[msae158-B58] Li H . Aligning sequence reads, clone sequences and assembly contigs with BWA-MEM. arXiv. 2013(1303.3997), preprint: not peer reviewed.

[msae158-B59] Li H, Durbin R. Fast and accurate short read alignment with Burrows–Wheeler transform. Bioinformatics. 2009:25(14):1754–1760. 10.1093/bioinformatics/btp324. 19451168 PMC2705234

[msae158-B60] Li X, Yang J, Shen M, Xie X-L, Liu G-J, Xu Y-X, Lv F-H, Yang H, Yang Y-L, Liu C-B, et al Whole-genome resequencing of wild and domestic sheep identifies genes associated with morphological and agronomic traits. Nat Commun. 2020:11(1):2815. 10.1038/s41467-020-16485-1. 32499537 PMC7272655

[msae158-B61] Lösch S, Grupe G, Peters J. Stable isotopes and dietary adaptations in humans and animals at pre-pottery Neolithic Nevallı Çori, southeast Anatolia. Am J Phys Anthropol. 2006:131(2):181–193. 10.1002/ajpa.20395. 16596597

[msae158-B62] Lv FH, Cao YH, Liu GJ, Luo LY, Lu R, Liu MJ, Li MH, Zhou P, Wang X-H, Shen M, et al Whole-genome resequencing of worldwide wild and domestic sheep elucidates genetic diversity, introgression, and agronomically important loci. Mol Biol Evol. 2022:39(2):msab353. 10.1093/molbev/msab353. 34893856 PMC8826587

[msae158-B63] Lv F-H, Peng W-F, Yang J, Zhao Y-X, Li W-R, Liu M-J, Ma Y-H, Zhao Q-J, Yang G-L, Wang F, et al Mitogenomic meta-analysis identifies two phases of migration in the history of eastern Eurasian sheep. Mol Biol Evol. 2015:32(10):2515–2533. 10.1093/molbev/msv139. 26085518 PMC4576706

[msae158-B64] Machová K, Málková A, Vostrý L. Sheep post-domestication expansion in the context of mitochondrial and Y chromosome haplogroups and haplotypes. Genes (Basel). 2022:13(4):613. 10.3390/genes13040613. 35456419 PMC9025449

[msae158-B65] Marciniak A . The secondary products revolution: empirical evidence and its current zooarchaeological critique. J World Prehist. 2011:24(2-3):117–130. 10.1007/s10963-011-9045-7.

[msae158-B66] McKenna A, Hanna M, Banks E, Sivachenko A, Cibulskis K, Kernytsky A, Garimella K, Altshuler D, Gabriel S, Daly M, et al The Genome Analysis Toolkit: a MapReduce framework for analyzing next-generation DNA sequencing data. Genome Res. 2010:20(9):1297–1303. 10.1101/gr.107524.110. 20644199 PMC2928508

[msae158-B67] Meadows JR, Cemal I, Karaca O, Gootwine E, Kijas JW. Five ovine mitochondrial lineages identified from sheep breeds of the near East. Genetics. 2007:175(3):1371–1379. 10.1534/genetics.106.068353. 17194773 PMC1840082

[msae158-B68] Meyer M, Kircher M. Illumina sequencing library preparation for highly multiplexed target capture and sequencing. Cold Spring Harb Protoc. 2010:2010(6):pdb.prot5448. 10.1101/pdb.prot5448. 20516186

[msae158-B69] Middleton C . The beginning of herding and animal management: the early development of caprine herding on the Konya plain, central Anatolia. Anatol Stud. 2018:68:1–31. 10.1017/S0066154618000017.

[msae158-B70] Mittnik A, Wang C-C, Svoboda J, Krause J. A molecular approach to the sexing of the triple burial at the Upper Paleolithic Site of Dolní Věstonice. PLoS One. 2016:11(10):e0163019. 10.1371/journal.pone.0163019. 27706187 PMC5051676

[msae158-B71] Morell Miranda P . 2023. Following the herd: population genetics of European sheep in time and space [Doctoral dissertation, Uppsala Universitet]. Uppsala: Acta Universitatis Upsaliensis, p. 76.

[msae158-B72] Nadler C, Hoffmann R, Woolf A. G-band patterns as chromosomal markers, and the interpretation of chromosomal evolution in wild sheep (*Ovis*). Experientia. 1973:29(1):117–119. 10.1007/BF01913288. 4125751

[msae158-B73] Naval-Sanchez M, Nguyen Q, McWilliam S, Porto-Neto LR, Tellam R, Vuocolo T, Reverter A, Perez-Enciso M, Brauning R, Clarke S, et al Sheep genome functional annotation reveals proximal regulatory elements contributed to the evolution of modern breeds. Nat Commun. 2018:9(1):859. 10.1038/s41467-017-02809-1. 29491421 PMC5830443

[msae158-B74] Niemi M, Bläuer A, Iso-Touru T, Nyström V, Harjula J, Taavitsainen J-P, Storå J, Lidén K, Kantanen J. Mitochondrial DNA and Y-chromosomal diversity in ancient populations of domestic sheep (*Ovis aries*) in Finland: comparison with contemporary sheep breeds. Genet Sel Evol. 2013:45(1):1–14. 10.1186/1297-9686-45-2. 23339395 PMC3558444

[msae158-B75] Patterson N, Moorjani P, Luo Y, Mallick S, Rohland N, Zhan Y, Genschoreck T, Webster T, Reich D. Ancient admixture in human history. Genetics. 2012:192(3):1065–1093. 10.1534/genetics.112.145037. 22960212 PMC3522152

[msae158-B76] Patterson N, Price AL, Reich D. Population structure and eigenanalysis. PLoS Genet. 2006:2(12):e190. 10.1371/journal.pgen.0020190. 17194218 PMC1713260

[msae158-B77] Pedrosa S, Uzun M, Arranz J-J, Gutiérrez-Gil B, San Primitivo F, Bayón Y. Evidence of three maternal lineages in Near Eastern sheep supporting multiple domestication events. Proc Biol Sci. 2005:272(1577):2211–2217. 10.1098/rspb.2005.3204. 16191632 PMC1559946

[msae158-B78] Peters J, von den Dreisch A, Helmer D. The upper Euphrates-Tigris basin: cradle of agro-pastoralism? In: New methods and the first steps of mammal domestication. Proceedings of the 9th International Council of Archeozoology (Durham, 23rd–28th August 2002). Oxford: Oxbow Books; 2005. p. 96–123.

[msae158-B79] Poplin F . Origine du Mouflon de Corse dans une nouvelle perspective paléontologique: Par marronnage. Ann Genet Sel Anim. 1979:11(2):133–143. 10.1186/1297-9686-11-2-133. 22896133 PMC2718923

[msae158-B80] Portillo M, García-Suárez A, Matthews W. Livestock faecal indicators for animal management, penning, foddering and dung use in early agricultural built environments in the Konya Plain, Central Anatolia. Archaeol Anthropol Sci. 2020:12(2):40. 10.1007/s12520-019-00988-0. 32025271 PMC6977141

[msae158-B81] Price TD . Europe's first farmers. Cambridge: Cambridge University Press; 2000. 10.1017/CBO9780511607851.

[msae158-B82] Quinlan AR, Hall IM. BEDTools: a flexible suite of utilities for comparing genomic features. Bioinformatics. 2010:26(6):841–842. 10.1093/bioinformatics/btq033. 20110278 PMC2832824

[msae158-B83] Racimo F, Woodbridge J, Fyfe RM, Sikora M, Sjögren K-G, Kristiansen K, Vander Linden M. The spatiotemporal spread of human migrations during the European Holocene. Proc Natl Acad Sci USA. 2020:117(16):8989–9000. 10.1073/pnas.1920051117. 32238559 PMC7183159

[msae158-B84] Ramsey CB . Bayesian analysis of radiocarbon dates. Radiocarbon. 2009:51(1):337–360. 10.1017/S0033822200033865.

[msae158-B85] R Core Team . R: a language and environment for statistial computing. Vienna, Austria: R Foundation for Statistical Computing; 2023.

[msae158-B86] Reimer PJ, Austin WE, Bard E, Bayliss A, Blackwell PG, Ramsey CB, Butzin M, Cheng H, Edwards RL, Friedrich M, et al The IntCal20 Northern Hemisphere radiocarbon age calibration curve (0–55 cal kBP). Radiocarbon. 2020:62(4):725–757. 10.1017/RDC.2020.41.

[msae158-B87] Sambrook J, Fritsch ER, Maniatis T. Molecular cloning: a laboratory manual. (2nd ed.). Cold Spring Harbor (NY): Cold Spring Harbor Laboratory Press; 1989.

[msae158-B88] Sanna D, Barbato M, Hadjisterkotis E, Cossu P, Decandia L, Trova S, Pirastru M, Leoni GG, Naitana S, Francalacci P, et al The first mitogenome of the Cyprus mouflon (*Ovis gmelini ophion*): new insights into the phylogeny of the genus *Ovis*. PLoS One. 2015:10(12):e0144257. 10.1371/journal.pone.0144257. 26636977 PMC4670089

[msae158-B89] Schoop U . Weaving society in Late Chalcolithic Anatolia: textile production and social strategies in the 4th millennium BC. In: Horejs B, Mehofer M, editors. Western Anatolia before troy. Proto-urbanisation in the 4th millennium BC? Vienna: Austrian Academy of Sciences Press; 2014. p. 421–446.

[msae158-B90] Schubert M, Lindgreen S, Orlando L. AdapterRemoval v2: rapid adapter trimming, identification, and read merging. BMC Res Notes. 2016:9(1):1–7. 10.1186/s13104-016-1900-2. 26868221 PMC4751634

[msae158-B91] Scott A, Reinhold S, Hermes T, Kalmykov AA, Belinskiy A, Buzhilova A, Berezina N, Kantorovich AR, Maslov VE, Guliyev F, et al Emergence and intensification of dairying in the Caucasus and Eurasian steppes. Nat Ecol Evolut. 2022:6(6):813–822. 10.1038/s41559-022-01701-6. PMC9177415 35393601

[msae158-B92] Shennan S . The first farmers of Europe: an evolutionary perspective. Cambridge: Cambridge University Press; 2018.

[msae158-B93] Sherratt A . Plough and pastoralism: aspects of the secondary products revolution. In: Hodder I, Isaac G, Hammond N, editors. Pattern of the past: Studies in honour of David Clarke. Cambridge: Cambridge University Press; 1981. p. 261–305.

[msae158-B94] Sherratt A . The secondary exploitation of animals in the Old World. World Archaeol. 1983:15(1):90–104. 10.1080/00438243.1983.9979887.

[msae158-B95] Skoglund P, Mallick S, Bortolini MC, Chennagiri N, Hünemeier T, Petzl-Erler ML, Salzano FM, Patterson N, Reich D. Genetic evidence for two founding populations of the Americas. Nature. 2015:525(7567):104–108. 10.1038/nature14895. 26196601 PMC4982469

[msae158-B96] Skoglund P, Malmström H, Omrak A, Raghavan M, Valdiosera C, Günther T, Hall P, Tambets K, Parik J, Sjögren K-G, *et al*. Genomic diversity and admixture differs for Stone-Age Scandinavian foragers and farmers. Science. 2014:344(6185):747–750. 10.1126/science.1253448. 24762536

[msae158-B97] Stiner MC, Buitenhuis H, Duru G, Kuhn SL, Mentzer SM, Munro ND, Pöllath N, Quade J, Tsartsidou G, Özbaşaran M. A forager–herder trade-off, from broad-spectrum hunting to sheep management at Aşıklı Höyük, Turkey. Proc Natl Acad Sci USA. 2014:111(23):8404–8409. 10.1073/pnas.1322723111. 24778242 PMC4060719

[msae158-B98] Stiner MC, Munro ND, Buitenhuis H, Duru G, Özbaşaran M. An endemic pathway to sheep and goat domestication at Aşıklı Höyük (Central Anatolia, Turkey). Proc Natl Acad Sci USA. 2022:119(4):e2110930119. 10.1073/pnas.2110930119. 35042793 PMC8795544

[msae158-B99] Tapio M, Marzanov N, Ozerov M, Ćinkulov M, Gonzarenko G, Kiselyova T, Murawski M, Viinalass H, Kantanen J. Sheep mitochondrial DNA variation in European, Caucasian, and Central Asian areas. Mol Biol Evol. 2006:23(9):1776–1783. 10.1093/molbev/msl043. 16782761

[msae158-B100] Taylor WT, Pruvost M, Posth C, Rendu W, Krajcarz MT, Abdykanova A, Brancaleoni G, Spengler R, Hermes T, Schiavinato S, et al Evidence for early dispersal of domestic sheep into Central Asia. Nat Hum Behav. 2021:5(9):1169–1179. 10.1038/s41562-021-01083-y. 33833423

[msae158-B101] Uerpmann HP . The ancient distribution of ungulate mammals in the Middle East: fauna and archaeolog. Sites in Southwest Asia and Northeast Africa. 2700. Wiesbaden: Reichert Verlag; 1987.

[msae158-B102] Valdiosera C, Günther T, Vera-Rodríguez JC, Ureña I, Iriarte E, Rodríguez-Varela R, Simões LG, Martínez-Sánchez RM, Svensson EM, Malmström H, et al Four millennia of Iberian biomolecular prehistory illustrate the impact of prehistoric migrations at the far end of Eurasia. Proc Natl Acad Sci USA. 2018:115(13):3428–3433. 10.1073/pnas.1717762115. 29531053 PMC5879675

[msae158-B103] Verdugo MP, Mullin VE, Scheu A, Mattiangeli V, Daly KG, Maisano Delser P, Hare AJ, Burger J, Collins MJ, Kehati R, et al Ancient cattle genomics, origins, and rapid turnover in the Fertile crescent. Science. 2019:365(6449):173–176. 10.1126/science.aav1002. 31296769

[msae158-B104] Vigne JD . The origins of animal domestication and husbandry: a major change in the history of humanity and the biosphere. C R Biol. 2011:334(3):171–181. 10.1016/j.crvi.2010.12.009. 21377611

[msae158-B105] Vigne JD, Carrere I, Briois F, Guilaine J. The early process of mammal domestication in the Near East: new evidence from the Pre-Neolithic and Pre-Pottery Neolithic in Cyprus. Curr Anthropol. 2011:52(S4):S255–S271. 10.1086/659306.

[msae158-B106] Vigne JD, Carrere I, Guilaine J. Unstable status of early domestic ungulates in the Near East. In: The neolithic of Cyprus. supplement 43. Athens (Greece): École Française d’Athèns, Bull Corr Héllenique; 2003. p. 239–251.

[msae158-B107] Vigne JD, Helmer D. Was milk a “secondary product” in the Old World Neolithisation process? Its role in the domestication of cattle, sheep and goats. Anthropozoologica. 2007:42(2):9–40.

[msae158-B108] Vigne JD, Zazzo A, Cucchi T, Carrère I, Briois F, Guilaine J. The transportation of mammals to Cyprus sheds light on early voyaging and boats in the Mediterranean Sea. Eurasian Prehist. 2014:10(1–2):157–176.

[msae158-B109] Wang Y, Nielsen R. Estimating population divergence time and phylogeny from single-nucleotide polymorphisms data with outgroup ascertainment bias. Mol Ecol. 2012:21(4):974–986. 10.1111/j.1365-294X.2011.05413.x. 22211450 PMC3951478

[msae158-B110] Wang D-F, Orozco-terWengel P, Li MH, Lv FH. Genomic analyses of Asiatic Mouflon in Iran provide insights into the domestication and evolution of sheep. bioRxiv. 2023(561316). 10.1101/2023.10.06.561316, 25 October 2023, preprint: not peer reviewed. PMC12164106 40514664

[msae158-B111] Wickham H . ggplot2: elegant graphics for data analysis. New York: Springer-Verlag; 2016. https://ggplot2.tidyverse.org.

[msae158-B112] Wilkin S, Ventresca Miller A, Fernandes R, Spengler R, Taylor WT-T, Brown DR, Reich D, Kennett DJ, Culleton BJ, Kunz L, et al Dairying enabled Early Bronze Age Yamnaya steppe expansions. Nature. 2021:598(7882):629–633. 10.1038/s41586-021-03798-4. 34526723 PMC8550948

[msae158-B113] Yurtman E, Özer O, Yüncü E, Dağtaş ND, Koptekin D, Çakan YG, Özkan M, Akbaba A, Kaptan D, Atağ G, et al Archaeogenetic analysis of Neolithic sheep from Anatolia suggests a complex demographic history since domestication. Commun Biol. 2021:4(1):1279. 10.1038/s42003-021-02794-8. 34773064 PMC8589978

[msae158-B114] Zeder MA . Domestication and early agriculture in the Mediterranean Basin: origins, diffusion, and impact. Proc Natl Acad Sci USA. 2008:105(33):11597–11604. 10.1073/pnas.0801317105. 18697943 PMC2575338

[msae158-B115] Zeder MA . Out of the Fertile Crescent: the dispersal of domestic livestock through Europe and Africa. Cambridge: Cambridge University Press; 2017.

