# Supplementary material for: The Population History of Domestic Sheep Revealed by Paleogenomes
Source: Mol Biol Evol. 2024 Oct 22;41(10):msae158. doi: 10.1093/molbev/msae158 (PMC11495565; doi:10.1093/molbev/msae158)
Supplement: msae158_Supplementary_Data [file msae158_supplementary_data.zip › Description of archaeological sites.pdf]

Description of archaeological sites

<u>Anatolia</u>

Bademağacı Höyük

Bademağacı Höyük is situated in the south of the Lakes District in southwest Turkey, north of Antalya near the town of Bademağacı. The occupation of the site starts from the Early Neolithic period. A Middle Bronze Age occupation that continued for a while is noticeable. There is also a small church from the Early Christianity Period (Duru and Umurtak, 2019). The mammalian fauna of the Neolithic period consists of pigs (17%), sheep and goats (51%), and cattle (23%). Most of the mammalian fauna is composed of caprines, with sheep outnumbering goats. The small size of the postcranial bones of sheep has been considered indicative of the presence of domestic forms. Wild forms of sheep have also been reported in the zooarchaeological assemblage of the site (De Cupere et al., 2008). While culling profiles of sheep/goats point towards their breeding for milk exploitation at this site, the analysis of organic residues in potsherds indeed indicated the processing of ruminant fats, most likely the dairy products of sheep (De Cupere et al., 2019).

Barcın Höyük

The site is in the province of Bursa in northwestern Turkey. The site was occupied intermittently between the Neolithic and Byzantine periods (Gerritsen and Özbal, 2019). Neolithic levels of the site represent the earliest farming communities in the Marmara region (Gerritsen and Özbal, 2019; Özbal and Gerritsen, 2019). The initial settlers who arrived in the region around 6600 BCE, brought the domesticated crops and animals with them (Gerritsen et al., 2013a; Gerritsen et al., 2013b). While goat herding was a minor part of animal exploitation, mainly sheep and cattle were preferred as herd animals. Dairy consumption was common according to residue analysis on pottery from the Neolithic settlement layers.

Erbaba Höyük

The small Pottery Neolithic village of Erbaba is located in the Beyşehir region of Lakes District Anatolia. The site was occupied between 6700 and 6400 BCE. The assemblage was dominated by domestic caprines and cattle. Caprines were the most abundant fauna on all levels, with sheep outnumbering goat specimens. Sheep exhibit a wide range of variability in size considering the measurements of various bones (Arbuckle, 2006). This suggests that both wild sheep were hunted and consumed by the inhabitants, together with smaller, morphologically domestic animals as a separately managed population. It has also been speculated that hunting not only continued but even displaced herding as the dominant mode of caprine exploitation in the last phase of occupation (Arbuckle, 2008a, 2008b).

Pınarbaşı

Pınarbaşı is located on the eastern edge of the southwest Konya basin in Central Anatolia. The site consists of a rock shelter and adjacent slopes with a small archaeological mound on a promontory projecting west of the rock shelter area. The rock shelter has deep occupation deposits including those of the Epipalaeolithic dated c. 14,200 - 11,000 cal BC (Baird et al., 2013; Table 1). The rock shelter also has an overlying occupation dated to the Late Neolithic c. 6500 - 6000 cal BC. The small mound has early Holocene occupation dated c. 9500 - 7800 cal BC (Baird et al., 2018). Epipalaeolithic deposits are typified by thin ashy lenses interspersed with rock face debris lenses and occasional hearths. Overall in the

Epipalaeolithic regularly repeated but short term episodes of occupation are suggested over the period indicated by the C14 dates (Baird, 2023). In the Epipaleolithic period, the most abundant hunted animals were caprines making up 35% of the total number of identified species, with more of the diagnostic fragments being sheep (Baird et al., 2013). Aurochs and equids were also hunted and probably also made an important contribution to the diet along with fish, water birds, hare, tortoises, and possibly frogs (Baird, 2023). Canid remains were also common at the site. The Epipalaeolithic sheep sample whose results are discussed in this paper came from the later phases of the Epipalaeolithic occupation dated approximately in the period 11,500 - 11,000 cal BC.

Suberde Höyük

The site is in the Beyşehir-Suğla region of the Lakes District of Turkey. The occupation of the site dates between the Neolithic to Islamic periods, with Neolithic settlement occurring between 7500-6900 BCE. The remains of the caprines among the mammalian fauna are dominant with 81.5% of the identified remains. Sheep reportedly outnumbered the goats, which is a typical Anatolian Neolithic pattern (Peters et al., 1999; Martin et al., 2002; Russell and Martin, 2005; Arbuckle, 2006; Arbuckle and Ozkaya, 2006). Due to the absence of a young male kill-off pattern, it has been suggested that sheep management in Suberde did not follow the typical management pattern, but rather an "experimental exploitation system" (Arbuckle 2008a).

Tepecik-Çiftlik Höyük

Tepecik-Çiftlik is located in the Cappadocia region of Central Anatolia. It was occupied between 7100 and 5800 BCE, from the end of the Aceramic Neolithic until the early Chalcolithic period (Bıçakçı et al.,2012; Bıçakçı, 2022). Agriculture and animal exploitation are present starting from the Pottery Neolithic levels, together with continued hunting and gathering. Among the mammalian fauna at the site, 85% is composed of caprine bones. Similar to other Anatolian Neolithic sites, sheep samples outnumber the goat samples. Due to smaller females among the sheep samples, it is assumed that herding was the predominant exploitation method in Tepecik-Çiftlik rather than hunting.

Ulucak Höyük

Ulucak Höyük, located 25 km east of İzmir in West Central Turkey, is a small mound covering an area of ca. 1 ha with 11 m of stratigraphic sequence. It was occupied from the Neolithic to the Byzantine period (Çilingiroğlu et al., 2012; Çevik, 2013). Neolithic occupation at the site is represented by Levels VI through IV, dated from 6850/30 to 5670 cal. BC (Çilingiroğlu et. al. 2012; Çevik and Abay, 2016; Çevik and Erdoğu, 2020). Thus, it represents one of the earliest sites with evidence of farming and animal husbandry in western Anatolia (Çakırlar, 2012). Morphologically domestic caprines were husbanded along with cattle and pigs from the beginning of the settlement. Caprine exploitation was stable throughout time, unlike the relative abundance of pigs and deer which increased in time. Sheep were more abundant than goats, possibly due to being more profitable to breed in the well-watered plain of the site (Pilaar Birch et al., 2019).

<u>Iran</u>

Tepe Khaleseh

Tepe Khaleseh is a small site located in Zanjan province near the city of Khorram Darreh in northwestern Iran. Archaeological excavations of the site began in 2009 (Valipour et al., 2013). The site was occupied in three phases related to the late Neolithic period. The animal remains consisted of fragmentary post-consumption assemblages. Approximately 13600 animal remains were counted in studied assemblages from the Trench V. Almost 9700 (~71%) pieces were identified anatomically and zoologically (Gręzak et al., 2010). The vast majority of the skeletal material was mammal bone. The mammal remains belonged to: Bovidae (domestic cattle), Caprinae (sheep and goat, domestic and wild), Susidae (probably domestic pig), Equidae (onager), Gazella sp., Carnivora, Lepus sp., Mustelidae, and Rodentia. Ovis/Capra is the most common taxon at the site (almost 90% of the identified skeletal material). It appears as though the majority of both species belonged to domesticated animals, which could not be confirmed given the fragmentary nature of the collection. Similar conclusions could be drawn in the case of the cattle and pig bones.

<u>Russia</u>

Zahanata

In 1976, a joint expedition of the Kalmyk Research Institute of Language, Literature, History, and Kalmyk State University examined the construction zone of the Sarpinsk irrigation system, covering the Sarpinsky and Maloderbetovsky regions of the Kalmyk Autonomous Soviet Socialist Republic. As a result of the survey, a large number of archaeological monuments were discovered, mainly mounds, as well as sites and settlements. The mound group selected for excavation was located in the Zahanata tract on the lands of the Arshan-Zelmensky state farm, in the Sarpinsky district, between the state farm named after V.I. Chapaev (Hanata village) and the second farm of the Arshan-Zelmensky state farm, on the western bank of the Hanata swamp. The mound group consisted of 50 mounds. In 1976, managed to excavate 13 of them. A total of 64 burials were studied, covering the period from the Yamnaya culture of the Bronze Age to the late Middle Ages. All mounds had hemispherical mounds, many had ditches. All mounds were excavated for demolition, leaving edges from 0.5 to 1 m wide, depending on the diameter of the mound. All mounds consisted of homogeneous brownish sandy loam. The filling of the ditches is dark gray in color and rich in humus. The backfill of the burial pits differed from the mound in its darker color and structure, and the mainland in the absence of carbonates.

<u>K5 B19</u>

Mound (Kurgan) № 5 was located 876 m southwest of the large mound with a class III triangulation sign, which is located on the headland of the Hanata swamp, 4 km west of the western outskirts of the village of Hanata. The dimensions of the embankment from north to south are 44 m, from west to east - 45 m, height 1.99 m. The top of the mound is broken by a large burglar pit measuring 4 x 3.50 m, depth of 0.70 m. In this regard, the central benchmark on the mound deliberately shifted to the east, and the edge to the north for ease of study. The mound was excavated with a bulldozer, leaving an edge 3 m wide. After fixing

the layers, the edge was removed. Judging by the layers, the mound embankment was built in two stages, apparently over a pit burial destroyed by a robber crater, and topped up a second time over pit burial No. 19. The top layer under the turf cover was a meter thick, it consisted of brownish loose sandy loam, the lower one was thick 0.99 m, consisted of dense light brownish sandy loam. Twenty-seven burials were examined in the mound and on the mainland. The shape and size of the burial pits located in the embankment were most often not traced. In the burial mound, fragments of ceramics, headstock and horse teeth, fragments of human bones, and a rim were found, part of the rim and handle were missing (jug height 23 cm, bottom diameter 9.6 cm, body - 18.3 cm, neck - 10 cm).

The burial is late nomadic. Burial № 19 is an inlet burial, discovered in the embankment of the mound, one meter from the center to the east, at a depth of 1.85 m from it. The skeleton of a buried man lay on his back, with his knees sharply bent upside down, which eventually fell. The arms were bent at the elbows, and the hands of both hands lay on the pelvis. The buried person is oriented to the east-northeast. Two small pieces of ocher were found near the skull of the buried person. Near the feet and knees of the buried man lay fragments of a bull's jaw. A bull's horn and head were found near the knees of the buried person. This burial, belonging to the Yamnaya culture of the Bronze Age, is the oldest in the mound.

France

Menneville « Derrière-le-Village »

Menneville "Derrière le Village", 20 km north of Reims, is the easternmost final Linear Pottery (LBK – Early Neolithic) settlement in the river Aisne Valley. Unlike the other LBK sites in this area, the settlement is surrounded by a large interrupted ditch, enclosing a surface area of 6.4 hectares. Rescue excavations on the eastern third of the site (1989-1990) uncovered eight LBK floor plans and sixteen burials, located either alongside the houses or clustered just south of the enclosure ditch. Further human remains were found in the ditch, varying from isolated bones to primary burials of sixteen individuals (Thevenet et al., 2023). Since 2013, research excavations have been carried out on the central and western parts of the LBK enclosure, providing a range of new evidence for the funerary use of the ditch.

These ditch burials are associated with placed deposits of cattle bones, including bucrania, caprine, and pig skeletons (Hachem, 2021). The bone studied in this article comes from a whole lamb less than five months old. Radiocarbon dates on human bone from the settlement and ditch burials include Lyon-3566 [SacA-5460] 6110 ± 30 BP, 5203-4947 cal BCE (st. 93); Lyon-3567 [SacA-5461] 6070 ± 30 BP, 5049-4857 cal BCE (st. 188); Lyon-3568 [SacA-5462] 6090 ± 30 BP, 5193-4938 cal BCE (st. 248); Lyon-3570 [SacA-5464] 6055 ± 30 BP, 5033-4850 cal BCE (st. 254).

Bucy-le-Long « le Fond du Petit Marais »

Bucy-le-Long "le Fond du Petit Marais", about 50 km from Reims, is a settlement from the Blicquy-Villeneuve-Saint Germain (BVSG) period (4900-4700 BC) comprising five

contemporary houses (Constantin et al., 1995). The village covers an area of about one hectare and the settlement is organized in two alignments along a north-south axis. The houses, built on poles, are flanked by lateral pits in which consumer waste is collected. The fauna is relatively rich, with domestic animals, especially cattle, predominating over wild animals (Bedault, 2012). The bones analyzed in this article come from house 300, where sheep and goats occupy the largest space.

REFERENCES

Arbuckle, B. S. (2006). *The evolution of sheep and goat pastoralism and social complexity in Central Anatolia* [Ph.D.]. https://www.proquest.com/docview/305336456/abstract/5DFC9F5859344A77PQ/1?sourcetype=Dissertations%20&%20Theses

Arbuckle, B. S., & Özkaya, V. (2006). Animal exploitation at Körtik Tepe: An early Aceramic Neolithic site in southeastern Turkey. *Paléorient*, *32*(2), 113–136.

Arbuckle, B. S. (2008a). Caprine exploitation at Erbaba Höyük: A pottery Neolithic village in central Anatolia. *MOM Éditions*, *49*(1), 345–365.

Arbuckle, B. S. (2008b). Revisiting Neolithic caprine exploitation at Suberde, Turkey. *Journal of Field Archaeology*, *33*(2), 219–236.

Baird, D., Asouti, E., Astruc, L., Baysal, A., Baysal, E., Carruthers, D., Fairbairn, A., Kabukcu, C., Jenkins, E., Lorentz, K., Middleton, C., Pearson, J., & Pirie, A. (2013). Juniper smoke, skulls and wolves' tails. The Epipalaeolithic of the Anatolian plateau in its South-west Asian context; insights from Pınarbaşı. *Levant*, *45*, 175–209. https://doi.org/10.1179/0075891413Z.00000000024

Baird, D. (2023). *Güneydoğu asya bağlaminda anadolu platosu'nun epipaleolitik Dönem'i: Pinarbaşi in M Kartal ed. Türkiye'de Paleolitik Çağ. Kazılar*, 159-186. Bilgin Kültür Sanat Şti Ltd.
Bıçakçı, E. (2012). Bıçakçı, E., M. Godon, Y. G. Çakan-Tepecik-Çiftlik-The Neolithic in Turkey-2012. *The Neolithic in Turkey*. https://www.academia.edu/38600614/B%C4%B1%C3%A7ak%C3%A7%C4%B1_E_M_Godon_Y_G_%C3%87akan_Tepecik_%C3%87iftlik_The_Neolithic_in_Turkey_2012

Bedault, L. (2012). *L'exploitation des ressources animales dans la société du Néolithique ancien du Villeneuve-Saint-Germain en Bassin parisien: synthèse des données archéozoologiques*(Doctoral dissertation, Université de Paris 1–Panthéon-Sorbonne).

Bıçakçı, E. (2022). A Conspectus on the Status of Tepecik-Çiftlik Excavation (Cappadocia) Intersite and Regional Outcomes and. *6000 BC: Transformation and Change in the Near East and Europe*, 83.

Çakırlar, C. (2012). The evolution of animal husbandry in Neolithic central-west Anatolia: The zooarchaeological record from Ulucak Höyük (c. 7040–5660 cal. BC, Izmir, Turkey). *Anatolian Studies*, *62*, 1–33.

Çevik, Ö. (2013). *Ulucak Höyük 2009-2011 yılı kazı çalışmaları*. *Kazı Sonuçları Toplantısı*, *34*(1), 143-158.

Çevik, Ö., & Abay, E. (2016). Neolithisation in Aegean Turkey: towards a more realistic reading. *Anatolian Metal*, *7*, 187-97.

Çevik, Ö., & Erdoğu, B. (2020). Absolute Chronology of Cultural Continuity, Change and Break in Western Anatolia Between 6850-5460 cal. BC: The Ulucak Höyük Case. *Mediterranean Archaeology & Archaeometry*, *13*(1).

Çilingiroğlu, A., Çevik, Ö., & Cilingiroglu, C. (2012). Ulucak Höyük: Towards understanding the early farming communities of Middle West Anatolia: The contribution of Ulucak. *The Neolithic in Turkey*, *4*, 139–175.

Constantin, C., Farruggia, J. P., & Guichard, Y. (1995). Deuxième partie: Bucy-Le-Long," le Fonds Du Petit Marais". *Revue archéologique de Picardie*, *1*(1), 16-23.

De Cupere, B., Duru, R., & Umurtak, G. (2008). Animal husbandry at the Early Neolithic to Early Bronze Age site of Bademağacı (Antalya province, SW Turkey): Evidence from the faunal remains. *MOM Éditions*, *49*(1), 367–405.

De Cupere, B., Baeten, J., & Devos, D. (2019). Milk production at Bademağacı (SW Turkey) during the Early Neolithic: Archaeozoological data and residue analysis combined. In *Excavations at Bademağacı Höyük – I. The Neolithic and Early Chalcolithic settlements* (pp. 221–231). Yayınları.

Duru, R., Umurtak, G. (2019). *Bademağacı Höyüğü Kazıları Neolitik ve Erken Kalkolitik Çağ Yerleşmeleri I / Excavations at Bademağacı Höyük The Neolithic and Early Chalcolithic Settlements).* İstanbul: Ege Yayınları.

Gerritsen, F. A., Özbal, R. D., & Thissen, L. (2013a). Barcın Höyük. The Beginnings of Farming in the Marmara Region. In M. Özdoğan, N. Basgelen, & P. Kuniholm (Eds.), *The Neolithic in Turkey. New Excavations and New Research. Vol. 5 Northwestern Turkey and Istanbul* (pp. 93–112). Art and Archaeology Publications.

Gerritsen, F. A., Özbal, R. D., & Thissen, L. (2013b). The Earliest Neolithic Levels at Barcın Höyük, Northwestern Turkey. *Anatolica*, *39*, 53–92. https://doi.org/10.2143/ANA.39.0.2990784
Gerritsen, F., & Özbal, R. (2019). Barcın Höyük, a seventh millennium settlement in the Eastern Marmara region of Turkey. *Documenta Praehistorica*, *46*, 58–67. https://doi.org/10.4312/dp.46.4

Grężak, A., Sołtysiak, A., Valipour, H. R., & Davoudi, H. (2010). Tepe Khaleseh (Iran), season 2009. *Bioarchaeology of the Near East*, *4*, 58–62.

Hachem, L. (2021). Sign-Objects Among Neolithic Faunal Remains, Visible Symbols. *Open Archaeology*, *7*(1), 1564–1581. https://doi.org/10.1515/opar-2020-0202

Martin, L., Russell, N. and Carruthers, D. (2002*). Animal remains from the central Anatolian Neolithic'. In: Gérard, F. and Thissen, L. eds.* The Neolithic of Central Anatolia: Internal Developments and External Relations during the 9th-6th Millennia cal BC. Istanbul: Ege Yayınları, pp. 193-206.

Özbal, R., & Gerritsen, F. (2019). Barcın Höyük in Interregional Perspective: An Initial Assessment. In A. Marciniak (Ed.), *Concluding the Neolithic* (pp. 287–305). Lockwood Press. https://doi.org/10.5913/87913.CN.13

Peters, J., von den Driesch, A., Helmer, D., & Saña Segui, M. (1999). Early Animal Husbandry in the Northern Levant. *Paléorient*, *25*(2), 27–48.

Pilaar Birch, S. E., Scheu, A., Buckley, M., & Çakırlar, C. (2019). Combined osteomorphological, isotopic, aDNA, and ZooMS analyses of sheep and goat remains from Neolithic Ulucak, Turkey. *Archaeological and Anthropological Sciences*, *11*(5), 1669–1681. https://doi.org/10.1007/s12520-018-0624-8

Russell, N., & Martin, L. (2005). The Çatalhöyük mammal remains. *Russell, N. and Martin, L. (2005) The Çatalhöyük Mammal Remains. In: Hodder, I., (Ed.) Inhabiting Catalhoyuk: Reports from the 1995-1999 Seasons. McDonald Institute Monographs Series: Excavation Reports (4). McDonald Institute for Archaeological Research, Cambridge, UK, Pp. 33-98. ISBN 9781902937229*.

Thevenet, C., Ilett, M., Hachem, L., Baillieu, M., Hamon, C., & Allard, P. (2023). Nouvelles explorations, nouvelles observations sur l'enceinte rubanée de Menneville" Derrière le Village"(Aisne).

Valipour, H. R., Davoudi, H., Mostafapour, I., & Gręzak, A. (2013). Tepe Khaleseh, a Late Neolithic Site in Zanjan Province. *The Neolithisation of Iran: The Formation of New Societies*, *3*, 147–177.
