# Supplementary material for: The Population History of Domestic Sheep Revealed by Paleogenomes
Source: Mol Biol Evol. 2024 Oct 22;41(10):msae158. doi: 10.1093/molbev/msae158 (PMC11495565; doi:10.1093/molbev/msae158)
Supplement: msae158_Supplementary_Data [file msae158_supplementary_data.zip › Kaptan et al Supplemental Notes.pdf]

Supplemental Note 1

Shotgun sequencing of the Anatolian sheep osteological material revealed that the vast majority had little to no aDNA preservation (median endogenous DNA = 0.04%), which was notably lower than Anatolian human skeletal material from the same period (e.g. Yaka et al. 2021). One reason for lower sheep aDNA preservation may be the deficiency of petrous bones and teeth in our Anatolian sheep sample, which was dominated by long bone samples. Heat exposure during meat/bone processing in archaeological times may be another culprit.

Supplemental Note 2

The Neolithic sheep samples from Anatolia were morphologically identified as smaller in size compared to their wild counterparts, suggesting that they were domestic sheep (Arbuckle, 2008a; Arbuckle, 2008b; De Cupere et al., 2008; Çakırlar, 2012). Meanwhile, it is generally assumed that wild sheep were absent during the European Holocene (Barbato et al., 2017). Accordingly, it appears safe to assume that all Holocene samples studied here would be domestic sheep and not a wild game.

REFERENCES

Arbuckle, B. S. (2008a). Caprine exploitation at Erbaba Höyük: A pottery Neolithic village in central Anatolia. *MOM Éditions*, *49*(1), 345–365.

Arbuckle, B. S. (2008b). Revisiting Neolithic caprine exploitation at Suberde, Turkey. *Journal of Field Archaeology*, *33*(2), 219–236.

Barbato, M., Hailer, F., Orozco-terWengel, P., Kijas, J., Mereu, P., Cabras, P., Mazza, R., Pirastru, M., & Bruford, M. W. (2017). Genomic signatures of adaptive introgression from European mouflon into domestic sheep. *Scientific Reports*, *7*(1), 7623.

Çakırlar, C. (2012). The evolution of animal husbandry in Neolithic central-west Anatolia: The zooarchaeological record from Ulucak Höyük (c. 7040–5660 cal. BC, Izmir, Turkey). *Anatolian Studies*, *62*, 1–33.

De Cupere, B., Duru, R., & Umurtak, G. (2008). Animal husbandry at the Early Neolithic to Early Bronze Age site of Bademağacı (Antalya province, SW Turkey): Evidence from the faunal remains. *MOM Éditions*, *49*(1), 367–405.

Yaka, R., Doğu, A., Kaptan, D., Dağtaş, N., Chyleński, M., Vural, K., ... & Somel, M. (2021). Ancient genomics in Neolithic central Anatolia and Çatalhöyük.
