# Supplementary material for: The Population History of Domestic Sheep Revealed by Paleogenomes
Source: Mol Biol Evol. 2024 Oct 22;41(10):msae158. doi: 10.1093/molbev/msae158 (PMC11495565; doi:10.1093/molbev/msae158)
Supplement: msae158_Supplementary_Data [file msae158_supplementary_data.zip › Supplementary Figures.pdf]

SUPPLEMENTARY FIGURES

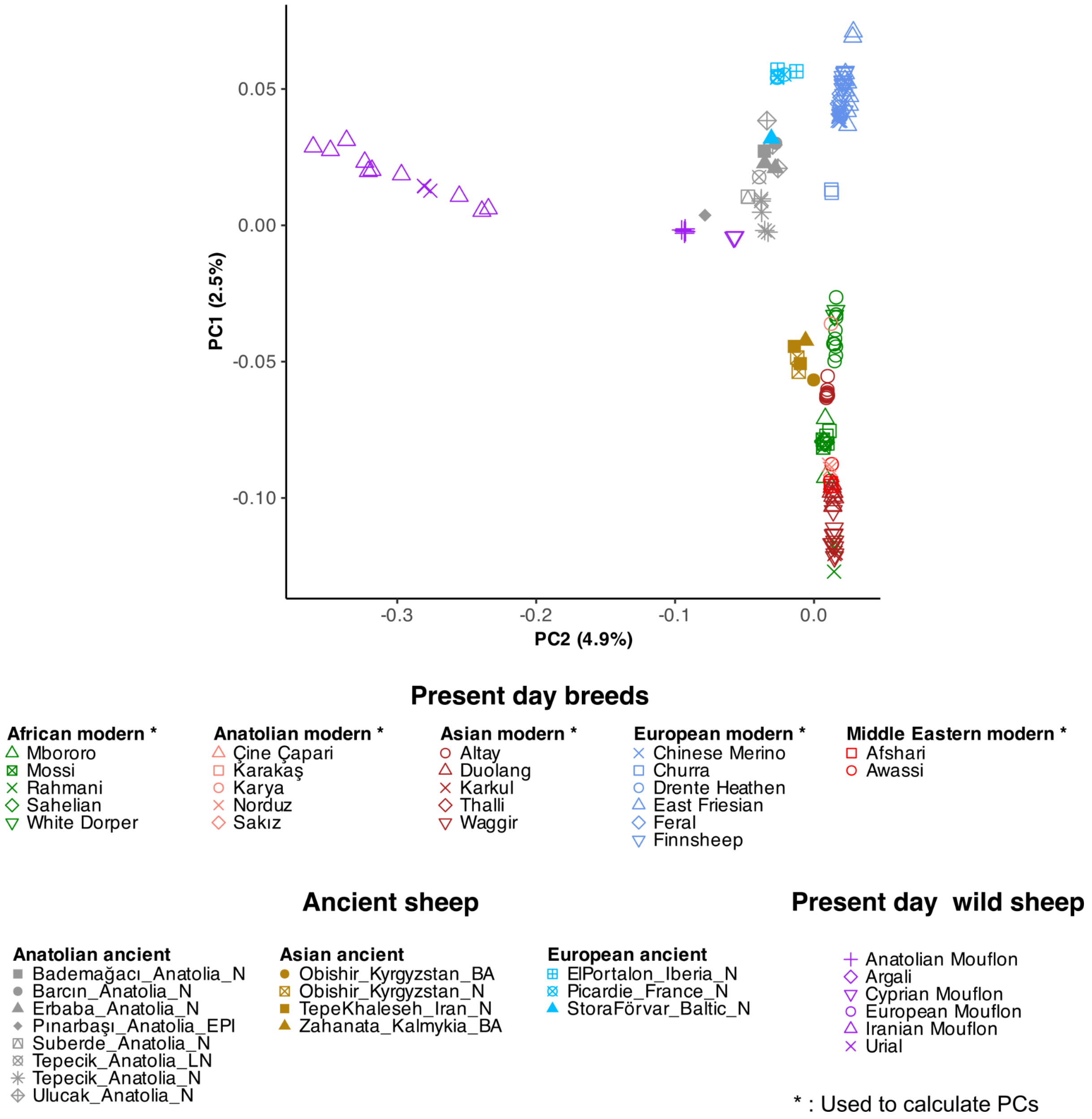

Supplementary Figure 1. **Principal component analysis (PCA) of genome-wide diversity without Argali population.** The genotype of each ancient individual was projected upon PC2 and PC3 calculated using 39 present-day sheep breeds (indicated with asterisks in the key, and in **Supplementary Table 2**) using the *de novo* SNP panel. The percentages on the x- and y-axes show the proportion of variance explained by PC2 and PC3, respectively.

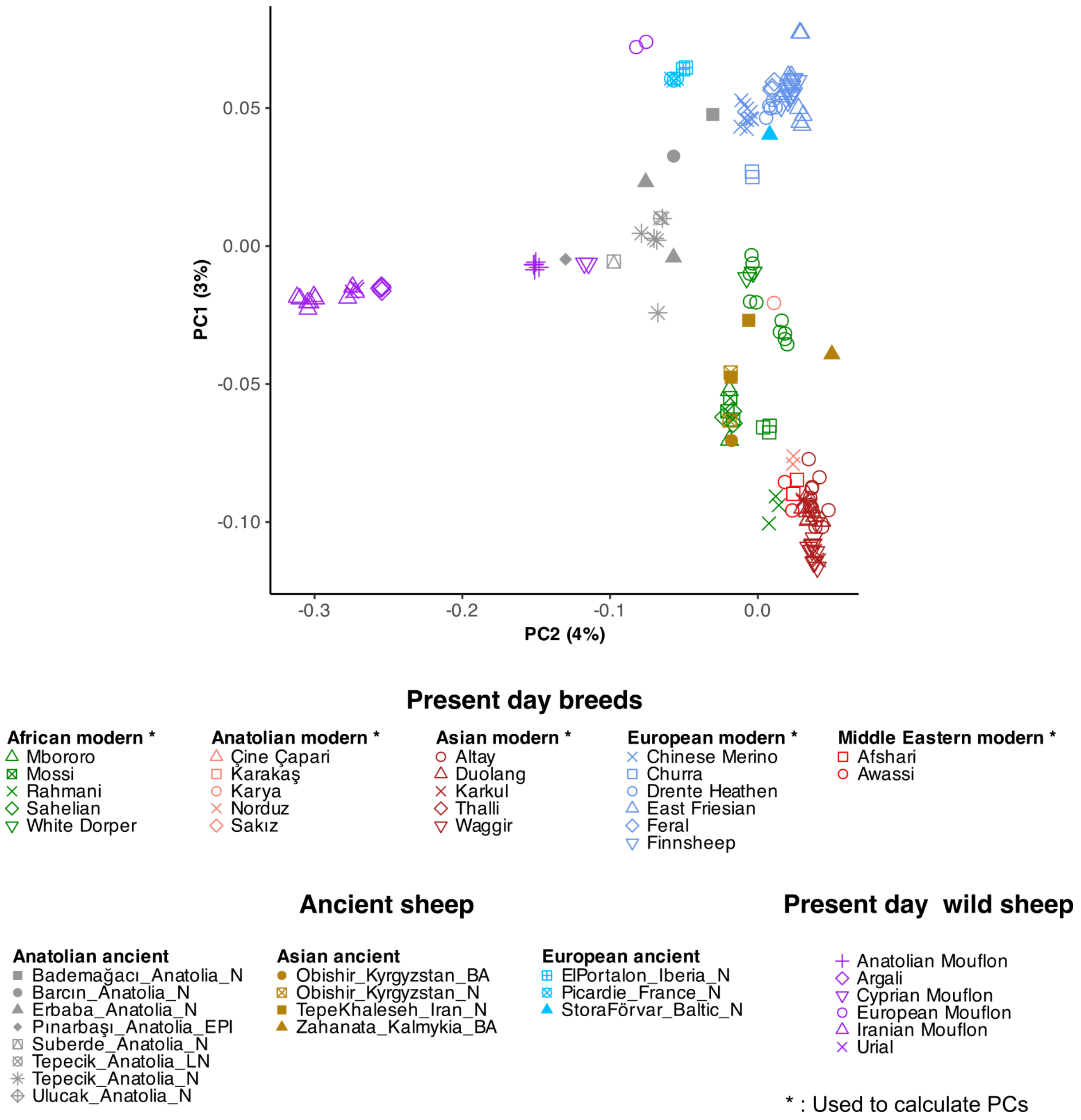

Supplementary Figure 2. **Principal component analysis (PCA) of genome-wide diversity with 50K SNP panel.** The genotype of each ancient individual was projected upon PC2 and PC3 calculated using 39 present-day sheep breeds (indicated with asterisks in the key, and in **Supplementary Table 2**) using the 50K SNP panel (Kijas et al., 2012). The percentages on the x- and y-axes show the proportion of variance explained by PC2 and PC3, respectively.

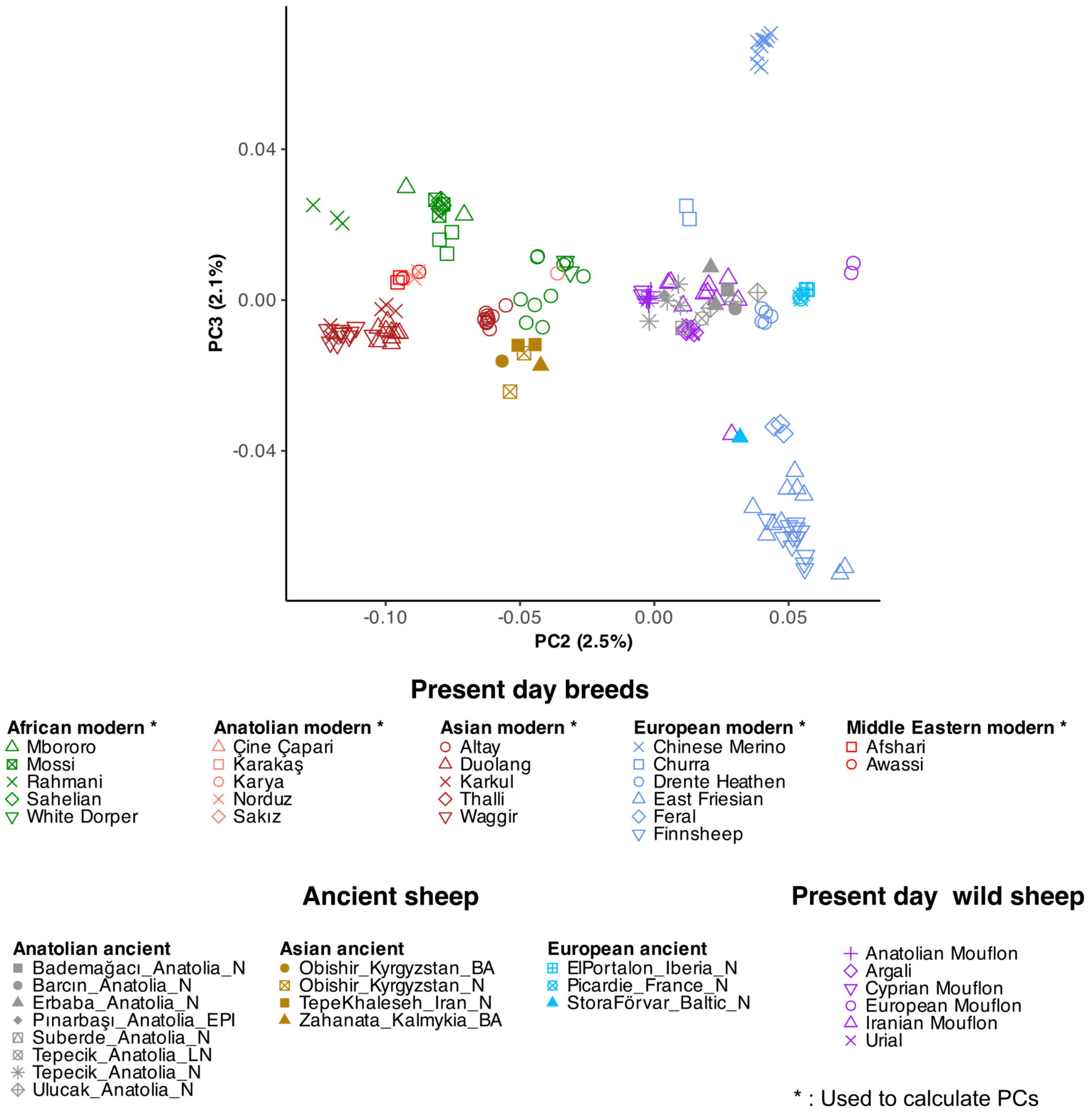

Supplementary Figure 3. **Principal component analysis (PCA) of genome-wide diversity.** PCA plot describes the genetic affinities among ancient and modern populations studied. The genotype of each ancient individual was projected upon PC2 and PC3 calculated using 39 present-day sheep breeds (indicated with asterisks in the key, and in **Supplementary Table 2**) using the *de novo* SNP panel. The percentages on the x- and y-axes show the proportion of variance explained by PC2 and PC3, respectively.

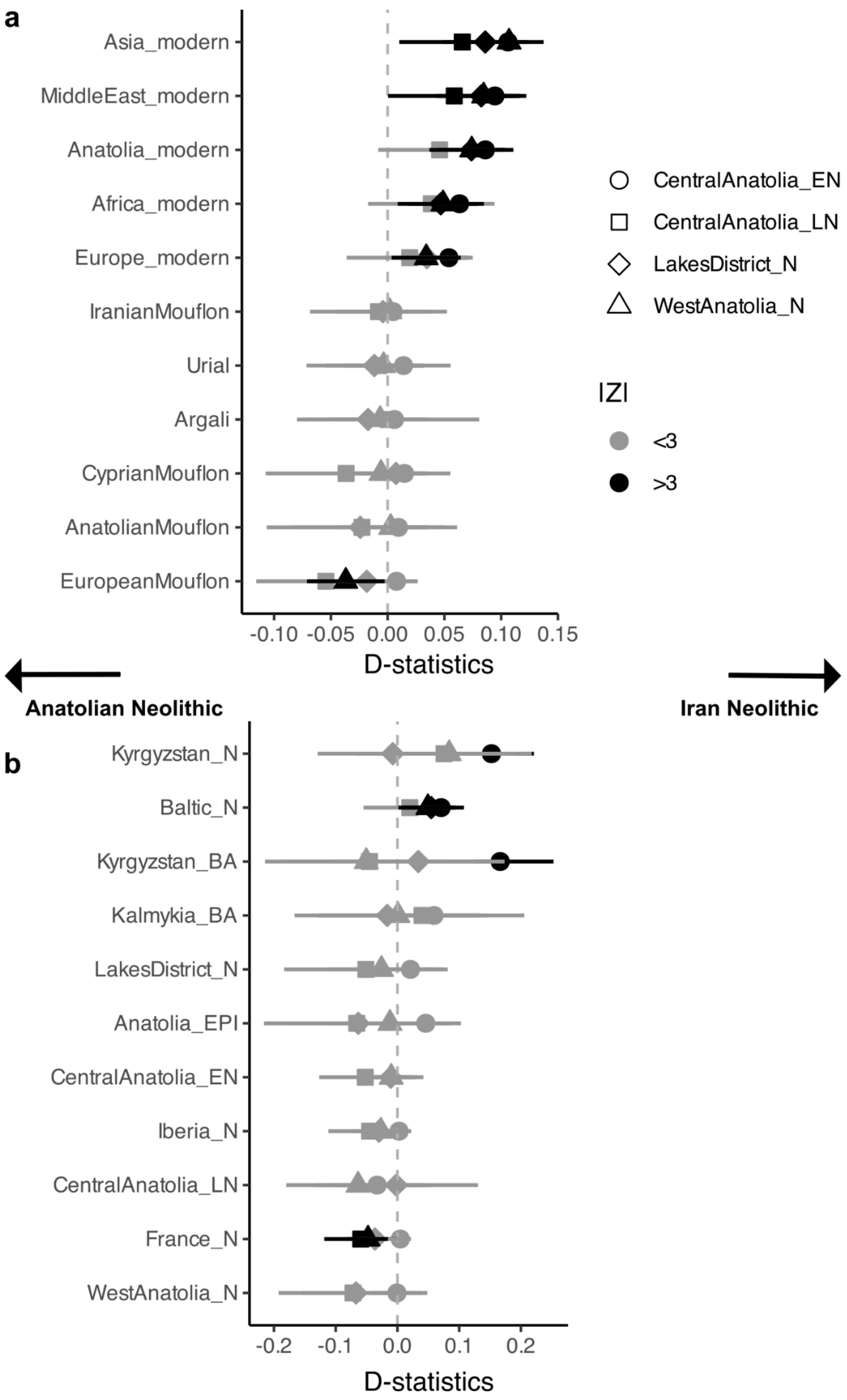

Supplementary Figure 4. D-statistics of Anatolian Neolithic Sheep (ANS) calculated as **(a)** *D(Goat, Modern sheep; ANS, Iran Neolithic)*. **(b)** *D(Goat, Ancient sheep; ANS, Iran Neolithic).* Error bars show ± 3 standard error.

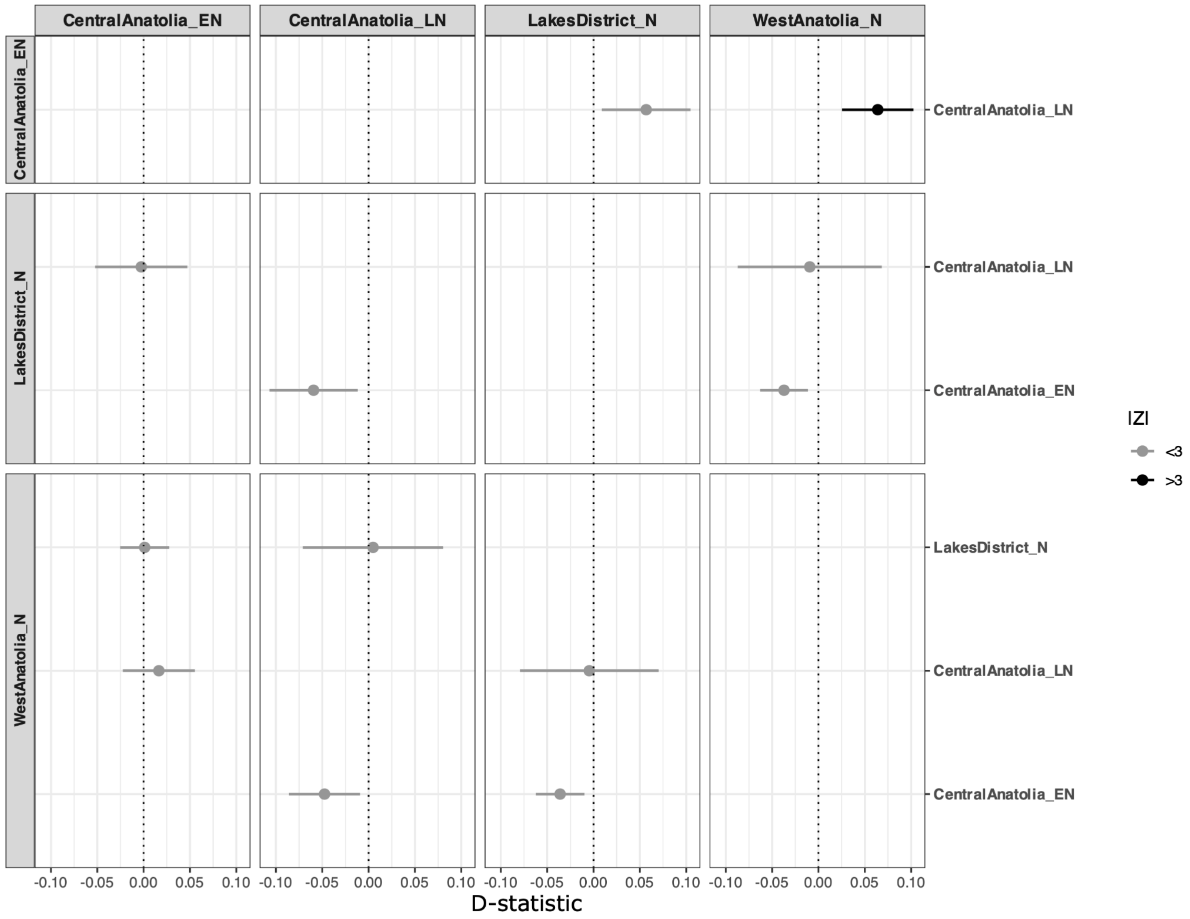

Supplementary Figure 5. D-statistics of Anatolian Neolithic Sheep (ANS) D-statistics of the form *D(Goat, ANS; ANS, ANS).*

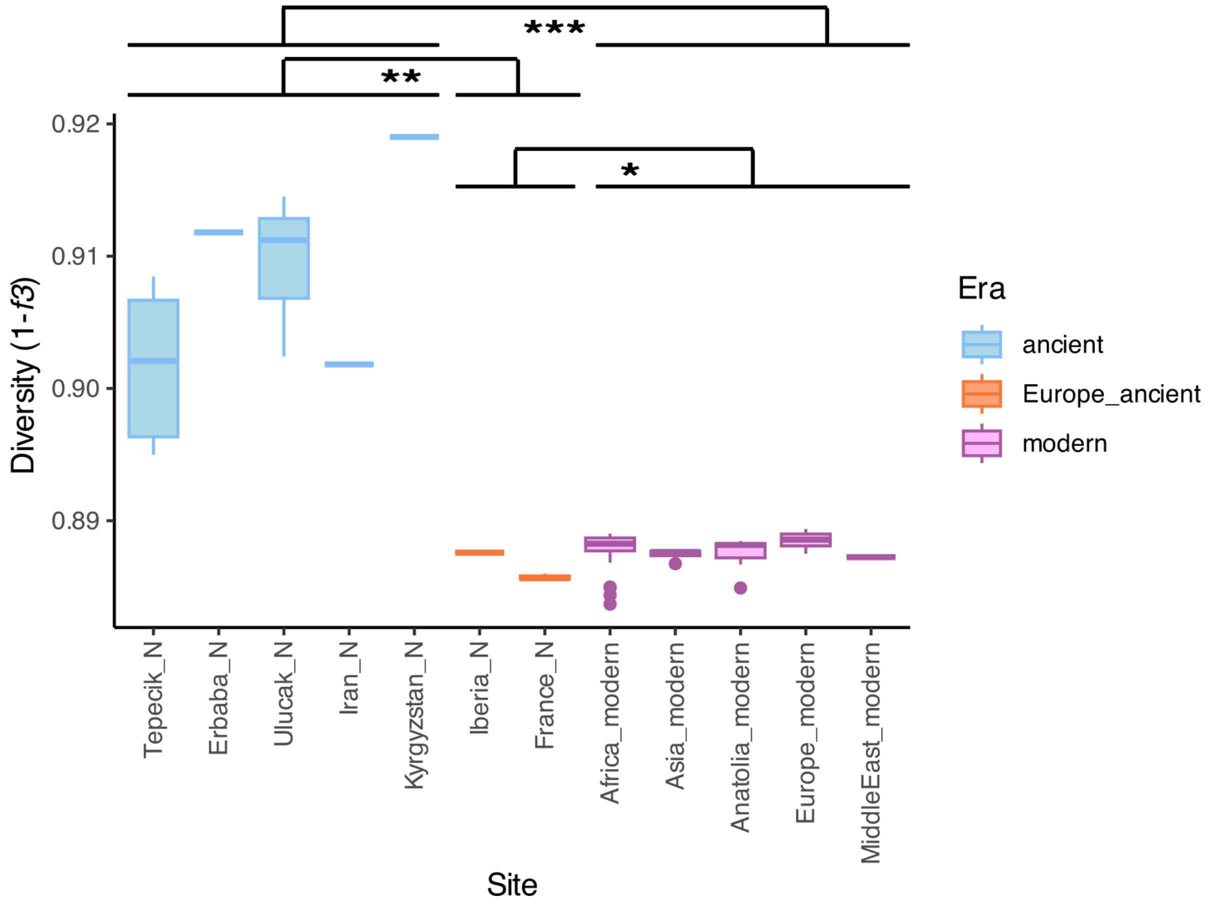

Supplementary Figure 6. Within-population genetic diversities are calculated using the (1-outgroup f_3) as a distance measure, shown on the y-axis. The x-axis depicts archaeological sites for ancient sheep populations (left) or regions of origin for modern-day sheep breeds (right). The within-population diversity was calculated using pairwise 1 - f_3 statistics with the outgroup-ascertained SNP panel with all SNPs. The boxplots show diversity measurements from more than two genomes per site. Cases shown with a single line include only two genomes. Sites with a single genome could not be included in this analysis. The 1 - f_3 values were used to compare diversities of Anatolia/Asia ancient, Europe ancient, and modern groups with the Kruskal-Wallis rank sum test (p= 5e-08). Between-group comparisons were further tested using Wilcoxon rank sum test ($p_{(Anatolia/Asia\ ancient\text{-}modern)}$= 8e-08, $p_{(Anatolia/Asia\ ancient\text{-}Europe\ ancient)}$=0.0011, $p_{(modern\text{-}Europe\ ancient)}$=0.0155).

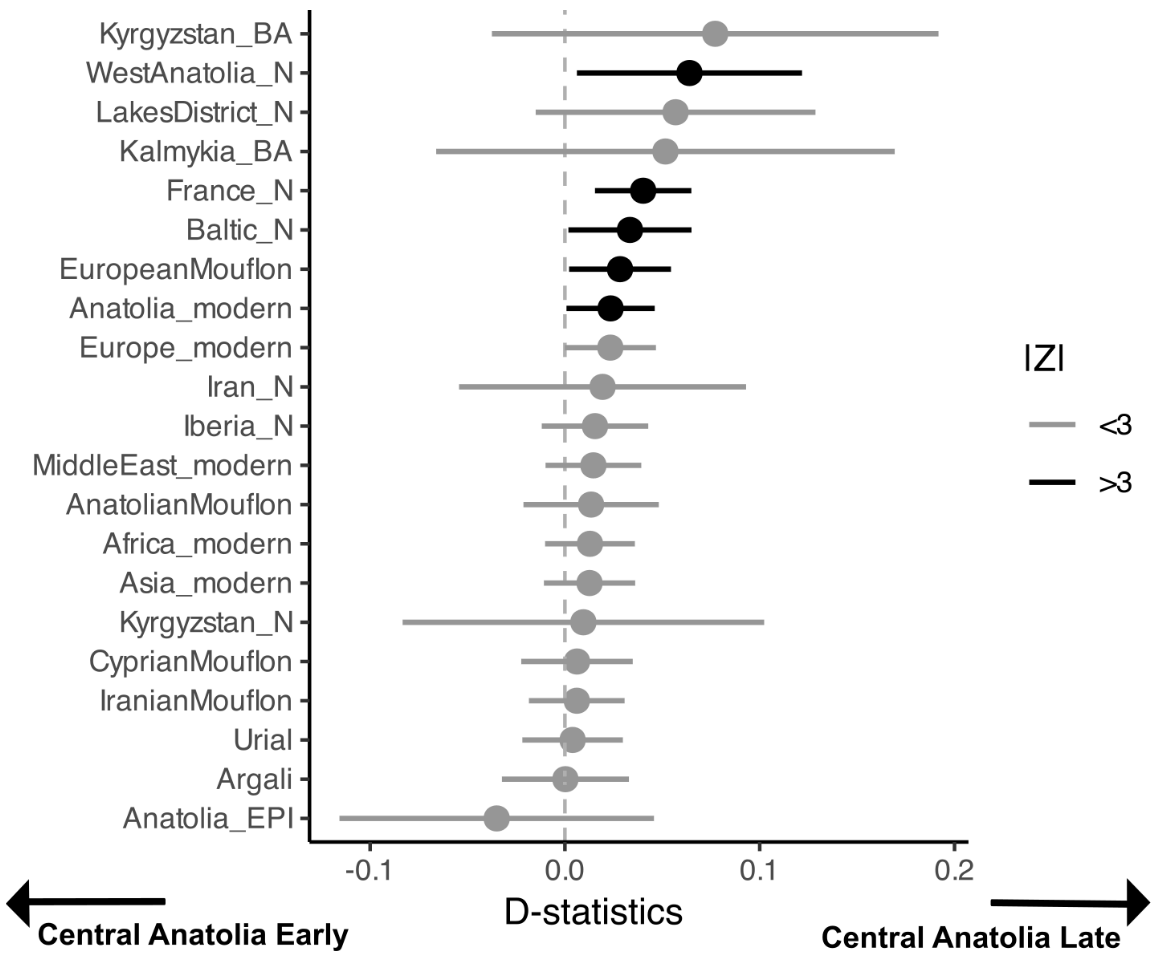

Supplementary Figure 7. ***D(Goat, X; Central Anatolia Early Neolithic, Central Anatolia Late Neolithic)***. **X**: All modern and ancient sheep. Error bars show ± 3 standard error.

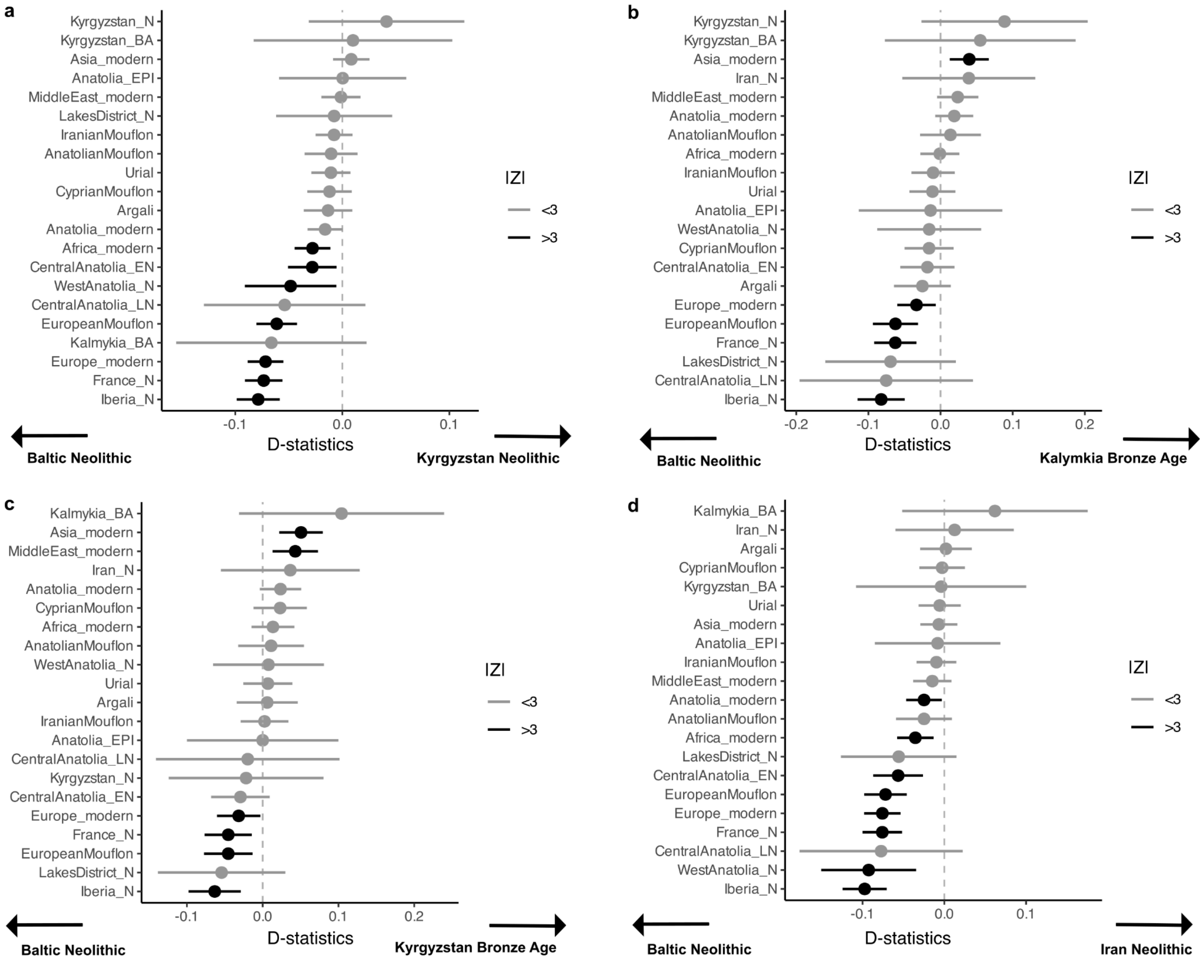

Supplementary Figure 8. ***D(Goat, X; Baltic Neolithic, Asian ancient)***. **(a)** *D(Goat, X; Baltic Neolithic, Kyrgyzstan Neolithic)* **(b)** *D(Goat, X; Baltic Neolithic, Kalmykia Bronze Age)* **(c)** *D(Goat, X; Baltic Neolithic, Kyrgyzstan Bronze Age)* **(d)** *D(Goat, X; Baltic Neolithic, Iran Neolithic),* **X**: All modern and ancient sheep. Error bars show ± 3 standard error.

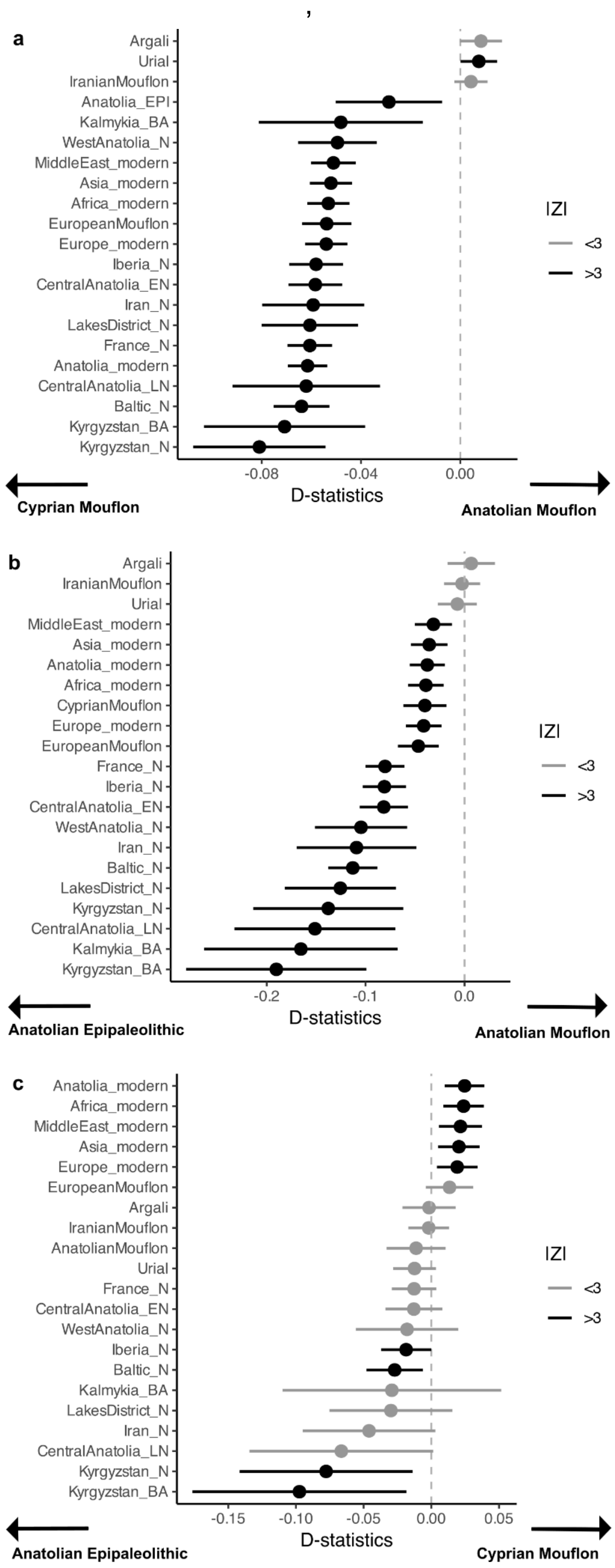

Supplementary Figure 9. **D-statistics in between Cyrpian Mouflon, Anatolian Mouflon and Anatolian Epipaleolithic. (a)** *D(Goat, X; Cyprian Mouflon, Anatolian Mouflon)* **(b)** *D(Goat, X; Anatolian Epipaleolithic, Anatolian Mouflon)* **(c)** *D(Goat, X; Anatolian Epipaleolithic, Cyprian Mouflon)*. **X**: All modern and ancient sheep. Error bars show ± 3 standard error.

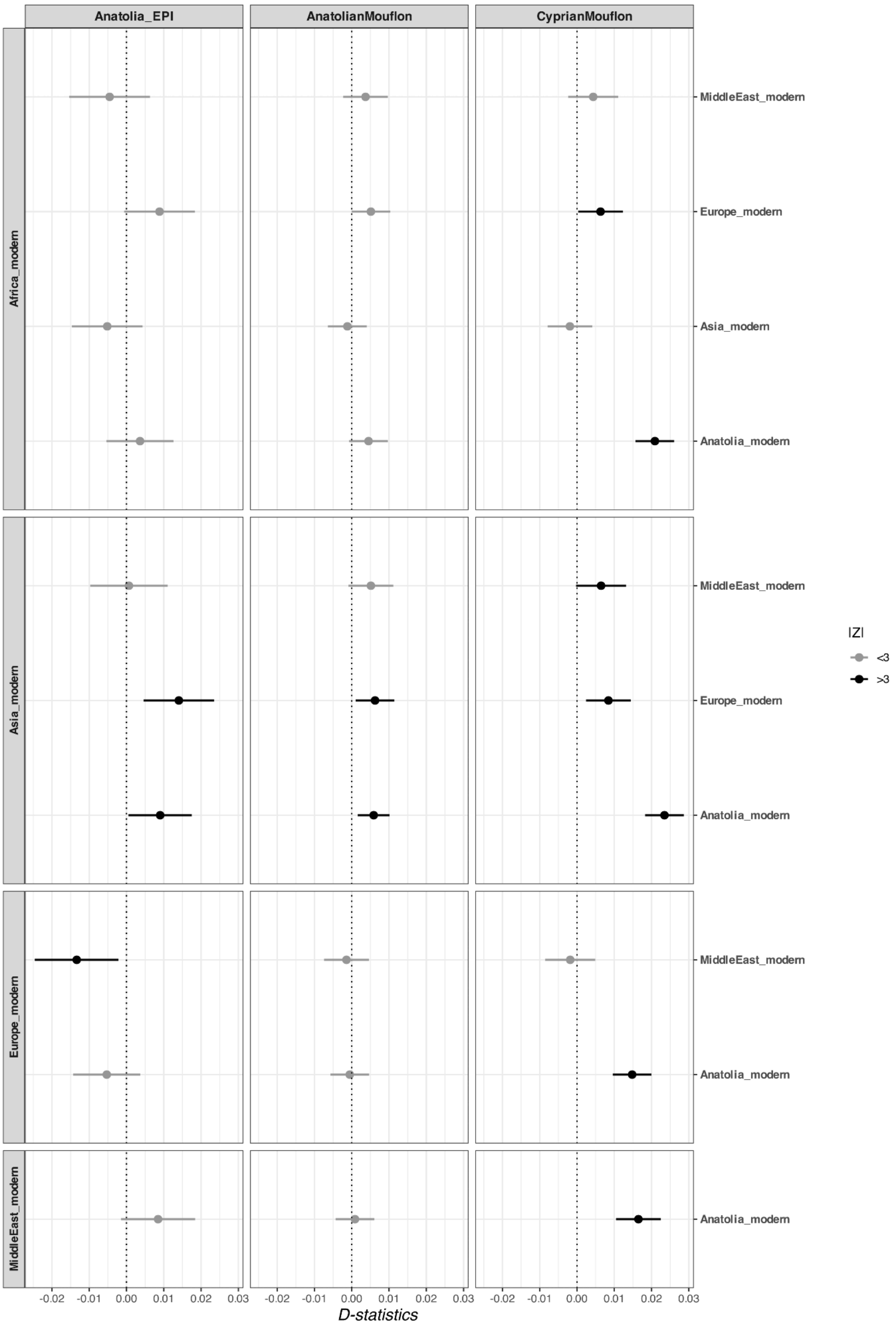

Supplementary Figure 10. ***D(Goat, Anatolian Mouflon/Cyprian Mouflon/Anatolian Epipaleolithic; Modern domestic sheep, Modern domestic sheep)***. **Modern domestic sheep**: Asia modern sheep, Africa modern sheep, Anatolia modern sheep, Middle East modern sheep. **Anatolian_EPI:** Anatolian Epipaleolithic. Error bars show ± 3 standard error.

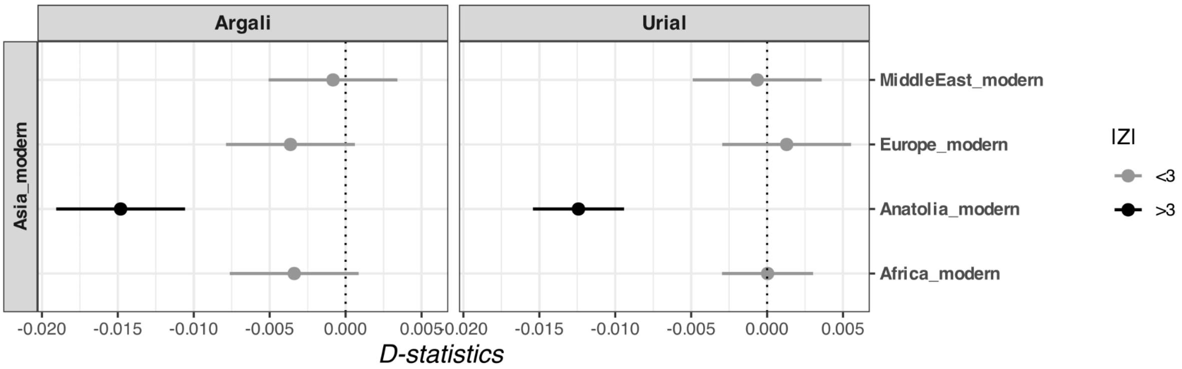

Supplementary Figure 11. ***D(Goat, Argali/Urial; Asia modern domestic, Modern domestic sheep)***. **Modern domestic sheep**: Europe modern sheep, Africa modern sheep, Anatolia modern sheep, Middle East modern sheep. Error bars show ± 3 standard error.
